# Beyond the Levant: First Evidence of a Pre-Pottery Neolithic Incursion into the Nefud Desert, Saudi Arabia

**DOI:** 10.1371/journal.pone.0068061

**Published:** 2013-07-19

**Authors:** Rémy Crassard, Michael D. Petraglia, Adrian G. Parker, Ash Parton, Richard G. Roberts, Zenobia Jacobs, Abdullah Alsharekh, Abdulaziz Al-Omari, Paul Breeze, Nick A. Drake, Huw S. Groucutt, Richard Jennings, Emmanuelle Régagnon, Ceri Shipton

**Affiliations:** 1 CNRS, Maison de l’Orient et de la Méditerranée, UMR 5133 ‘Archéorient’, Lyon, France; 2 School of Archaeology, Research Laboratory for Archaeology and the History of Art, University of Oxford, Oxford, United Kingdom; 3 Human Origins Program, Smithsonian Institution, Washington DC, United States of America; 4 Human Origins and Palaeoenvironments Research Group, Oxford Brookes University, Oxford, United Kingdom; 5 Centre for Archaeological Science, School of Earth and Environmental Sciences, University of Wollongong, Wollongong, New South Wales, Australia; 6 Department of Archaeology, College of Tourism and Archaeology, King Saud University, Riyadh, Saudi Arabia; 7 Ministry of Higher Education, Riyadh, Saudi Arabia; 8 The Saudi Commission for Tourism and Antiquities, Taif Antiquities Office, Taif, Saudi Arabia; 9 Department of Geography, King’s College London, London, United Kingdom; 10 School of Social Science, University of Queensland, Queensland, Australia; New York State Museum, United States of America

## Abstract

Pre-Pottery Neolithic assemblages are best known from the fertile areas of the Mediterranean Levant. The archaeological site of Jebel Qattar 101 (JQ-101), at Jubbah in the southern part of the Nefud Desert of northern Saudi Arabia, contains a large collection of stone tools, adjacent to an Early Holocene palaeolake. The stone tool assemblage contains lithic types, including El-Khiam and Helwan projectile points, which are similar to those recorded in Pre-Pottery Neolithic A and Pre-Pottery Neolithic B assemblages in the Fertile Crescent. Jebel Qattar lies ∼500 kilometres outside the previously identified geographic range of Pre-Pottery Neolithic cultures. Technological analysis of the typologically diagnostic Jebel Qattar 101 projectile points indicates a unique strategy to manufacture the final forms, thereby raising the possibility of either direct migration of Levantine groups or the acculturation of mobile communities in Arabia. The discovery of the Early Holocene site of Jebel Qattar suggests that our view of the geographic distribution and character of Pre-Pottery Neolithic cultures may be in need of revision.

## Introduction

**Figure 1:**
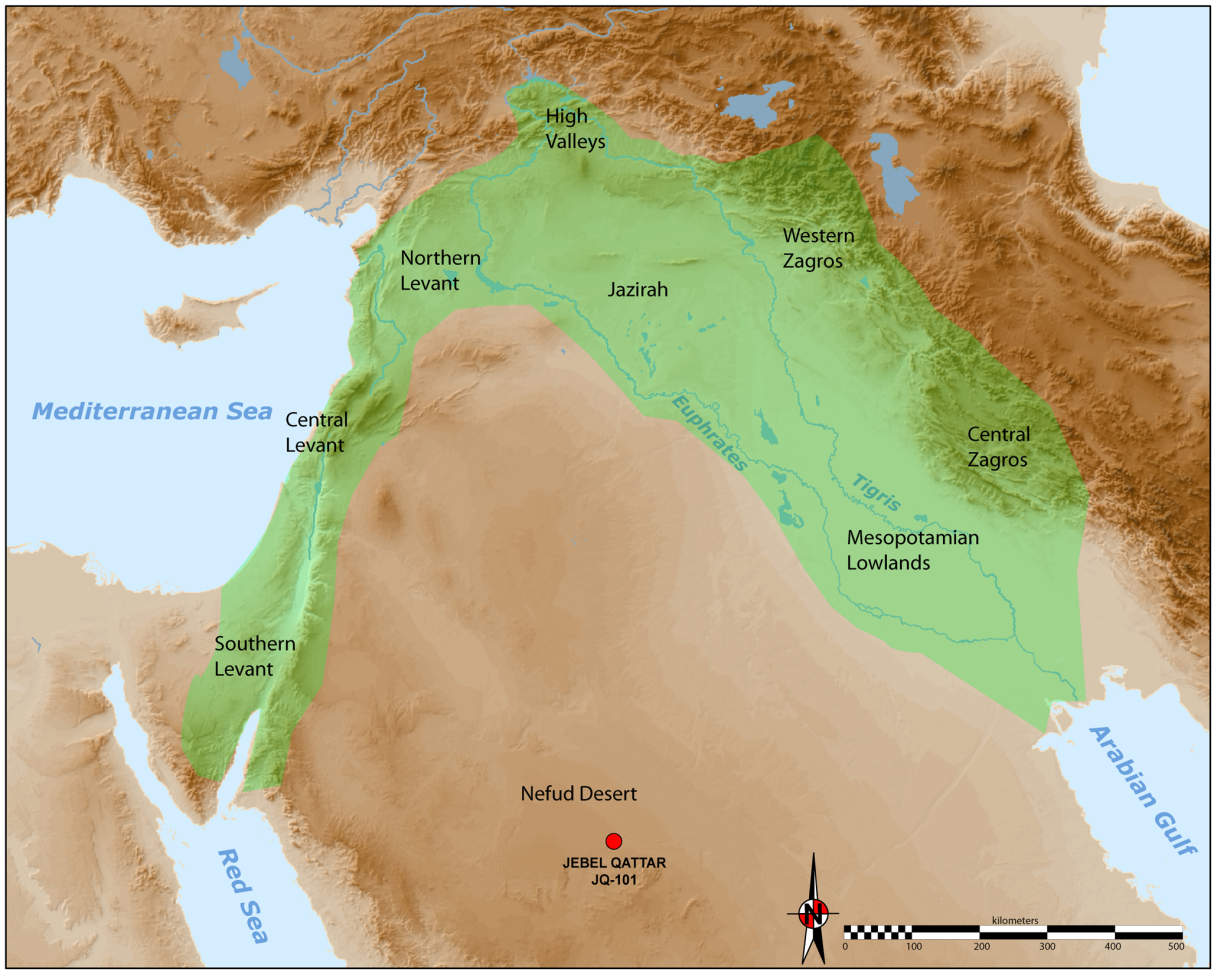
Map of the Neolithic Near East with the different geo-cultural zones of the core area (or Fertile Crescent), in green; after Aurenche and Kozlowski [82]. The JQ-101 site is located in the southern part of the Nefud Desert in Saudi Arabia.

The development of the Neolithic in Southwest Asia has long been seen as a pivotal phase in human evolution and history [1], [2]. To many scholars, a cultural and economic ‘revolution’ occurred in the Holocene, which fundamentally transformed the relationship between humans and their environments, paving the way for apopulation explosion, a shift towards sedentary settlement, and a profound change in technology. Most research attention has been focussed on the internal cultural dynamics of the ‘core area’ of the Fertile Crescent (see Figure 1. Less research has been devoted towards understanding the interactions between the core and periphery regions.

The processes which culminated in the ‘Neolithization’ of Southwest Asia have been much debated [3]–[9]. Debates have included the defining the origins of agriculture [10]–[15], the origins of animal domestication [16]–[20], the development of sedentism [21], [22], the evolution of proto-urban social systems [23], the demographic expansion of communities [24], [25], the genetic [26] and linguistic diversity [27] of groups, and the development of new beliefs and rituals in association with social changes [8], [28], [29]. A better understanding of the temporal and spatial and variability of early Neolithic cultures should also be viewed as a critical research task for archaeologists.

Archaeologists have identified two main components of the Neolithic of the Near East (Figure 1): (1) the aceramic Neolithic (Pre-Pottery Neolithic, or PPN), and (2) the Neolithic with ceramics (Pottery Neolithic, or PN).

The Pre-Pottery Neolithic A (PPNA: c. 10,300-9,600 BP *(Radiocarbon years before present (BP), where the ‘present’ is (by convention) defined as AD 1950)*/10,200-8,800 cal. BC; [30]) and the Pre-Pottery Neolithic B (PPNB: c. 9,600-8,600 BP/8,800-6,900 cal. BC; [30]) are two key periods for examining the first developmental stages of the Levantine Neolithic. The PPNA ranges over the Levant and the upper Mesopotamian region of the Fertile Crescent (often viewed as a “core area”), while the PPNB complex sites are located from central Anatolia to the Sinai (North-South) and from Cyprus to the Jazira (West-East). This classic chrono-cultural division of the Neolithic period, originally outlined by Kenyon [31], was based on stratigraphic investigations at the town of Jericho. The PPNA and PPNB periods are known to succeed the Natufian Epipalaeolithic culture and precede the ceramic Neolithic (PN). PPNA and PPNB archaeological sites typically feature the absence of pottery in a Neolithic context, with a variety of site types ranging from small circular mud brick dwellings to complex settled village communities, the cultivation of crops, especially cereals [32], [12], [33], and the hunting of wild game in association with the herding of sheep and goats [34], [35]. PPN communities also feature more elaborate burial customs [36], [37], architecture, subsistence economies and settlement types [38] foreshadowing the onset of proto-urbanism [39]. Little attention has been given to how PPN archaeological assemblages would look outside core areas of settlement, such as environments which were perhaps marginal for human occupation. Fuller et al. [14] outline the extent of PPNA ‘pre domestic cultivation’ across a broader area than the traditional ‘core’ region (contra Abbo et al. [40]). It is similarly probable that early Neolithic populations in Southwest Asia were temporally and spatially complex, and were perhaps interacting with surrounding populations who remained more classic hunter-gatherers.

PPNA and PPNB settlements of the Near East are also characterised on the basis of the wide and diverse range of lithic types that distinguish them from the preceding Upper Palaeolithic and Epipalaeolithic industries. Lithic typological [41], [42] and technological analyses have been conducted, including the analysis of reduction sequences and their dynamics (*chaînes opératoires*) of blank production, notably the study of blade manufacture on “naviform” cores [43]–[48].

**Figure 2:**
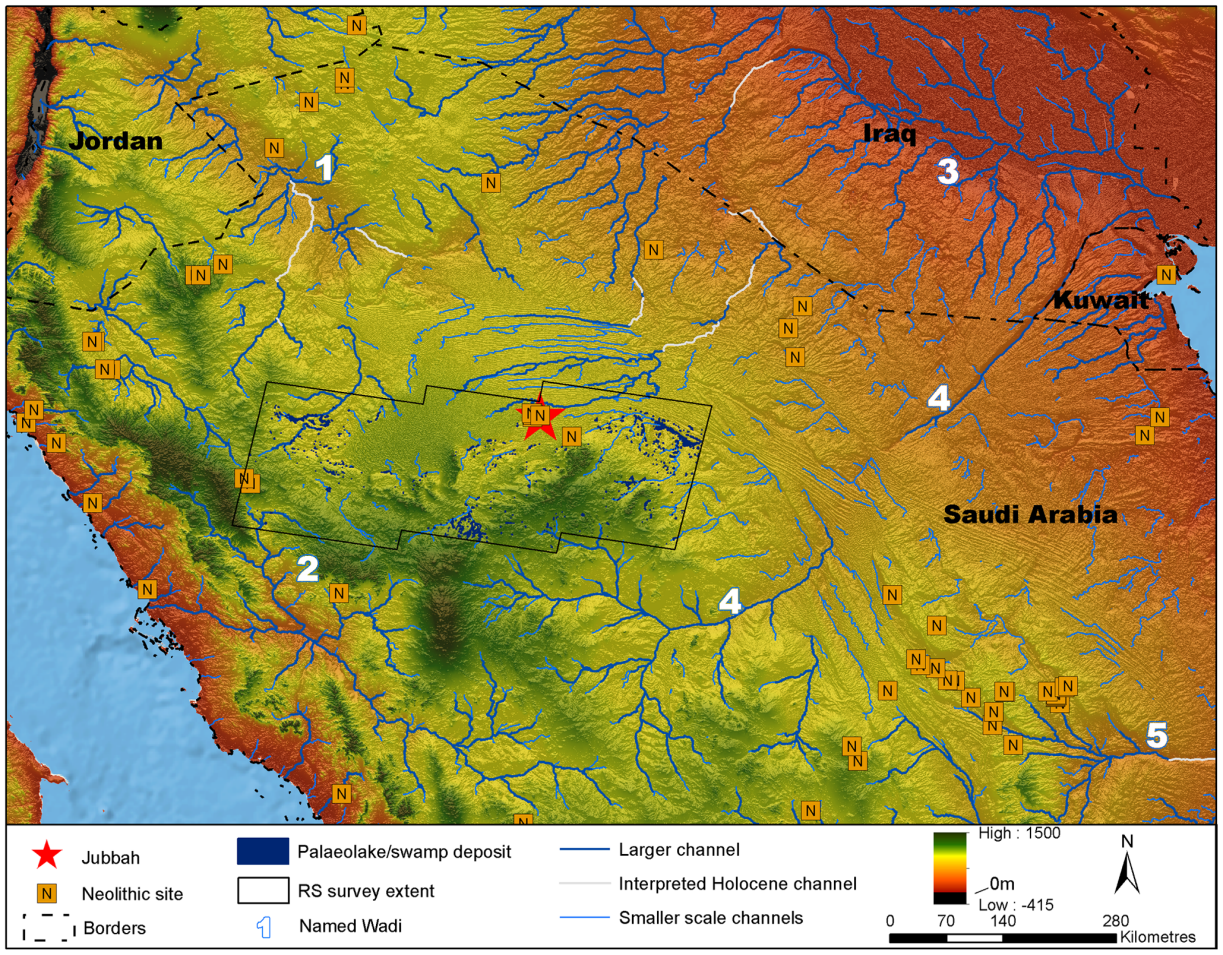
Neolithic sites of northern Arabia and palaeohydrology. Potential Holocene drainage is displayed in blue, with currently severed drainage connections that may have been active during Holocene humid periods interpreted and displayed in grey. Numbered Wadis: (1) Wadi as Sirhan, (2) Wadi al Hamd, (3) Euphrates, (4) Wadi al Batin, (5) Wadi Sabha. Potential palaeolake or swamp deposits detected through remote sensing which may relate to Holocene humidity are displayed for the region surrounding Jubbah. All data is overlain upon SRTMv4 elevation data [124] and Natural Earth 2 offshore data. Archaeological site locations calculated from survey data of the ‘Comprehensive Archaeological Survey Programme’, more information is provided in Groucutt and Petraglia [111].

As illustrated in Figure 2, a number of ‘Neolithic’ sites are known in northern Arabia [49]–[54]. These are virtually all surface sites, which have seen only cursory study. The presence of PPNA or PPNB technologies in the Arabian desert is only described in tentative notes [51]. We present here the first discovery of morphologically typical Levantine-like PPNA/PPNB stone tools, comprised mainly of diagnostic arrowheads, in the southern part of the Nefud Desert of northern Saudi Arabia. The recent discoveries at Jebel Qattar 101, Jubbah, represent the first clear occurrence of PPN lithic technologies in northern Arabia, about 500 kilometres south of the previously known distribution of PPN assemblages. Figure 2 shows how the distribution of putatively Neolithic sites in northern Arabia corresponds to the network of palaeolakes and palaeorivers in the region. The palaeodrainage in Figure 2 is derived from the HydroSHEDs dataset [55], as detailed in the supporting information for Petraglia et al. [56], whilst mapping of potential palaeolake and swamp deposits in the region around Jubbah was performed using the multispectral MF-SAM ratio outlined in Crassard et al. [57]. At present, palaeolakes have only been mapped in the region around Jubbah. In this region, palaeolacustrine sediment outcrops are patchy but abundant, which suggests that palaeolakes were locally abundant throughout the region and thus, that water was readily available during humid periods, feeding a well-developed lacustrine and riverine landscape that would have been conducive to human occupation and dispersal. Four large river systems (Figure 2; Wadi as Sirhan, Wadi al Hamd, the Euphrates and Wadi al Batin) have their headwaters in the vicinity of Jubbah and thus could have provided dispersal routes to the region. Of these, Wadi as Sirhan provides a likely dispersal route for PPN from the west and the Euphrates from the North. This notion is supported by presence of Neolithic sites throughout much of the region.

## Jebel Qattar 101 Lithic Site and Jebel Qattar 200 Lacustrine

### Site Jebel Qattar 101: General Description

The site of Jebel Qattar 101 (JQ-101) is situated near the modern town of Jubbah, approximately 350 km north of Riyadh. On the basis of archaeological discoveries reported in the vicinity of the Jubbah palaeolake [53], renewed archaeological and palaeoenvironmental surveys have recently been performed in the Jubbah palaeolake region in 2010 and 2011. Stratified Middle Palaeolithic sites have been reported occurring in wet phases of the Upper Pleistocene [56], [58]. Previous studies have also documented a wealth of rock art at Jubbah [51], [59], including suggestions that cattle, goats and other fauna depicted in the rock art represented the activities of Neolithic groups who were present in the Jubbah basin in the Early Holocene, although this is based on style alone, and not on any chronometric studies or archaeological investigations. A rock art survey was recently performed along several jebels in the region, including at the base of Jebel Qattar [60], resulting in the discovery of JQ-101. The JQ-101 site is so far a unique discovery, and notable for the large number of lithic artefacts distributed across a large surface area, measuring approximately 250×120 m, or 30,000 m^2^.

**Figure 3:**
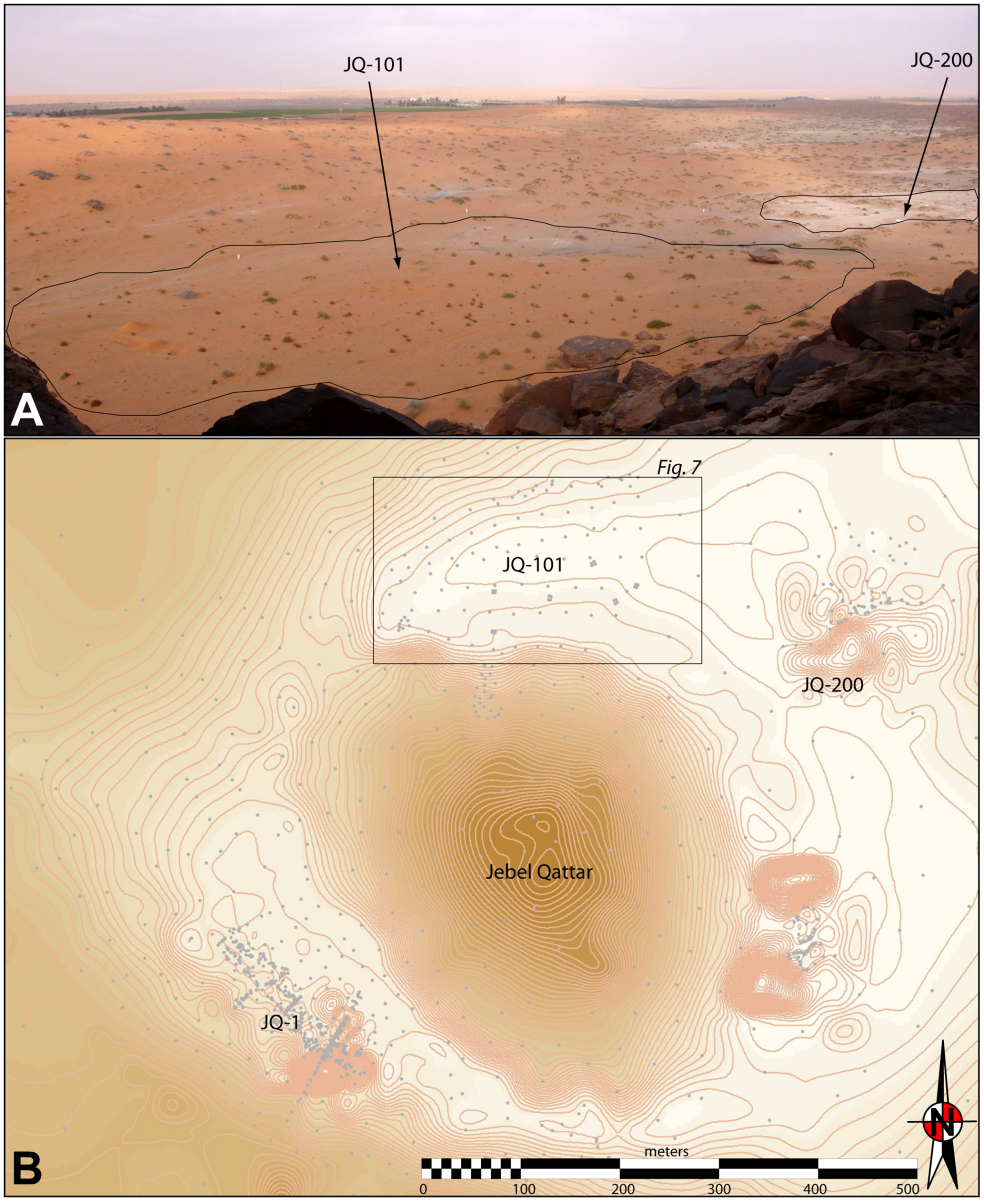
General setting of JQ-101 and JQ-200 at Jebel Qattar. A: 3D view of the sites, from the northern slope of Jebel Qattar; B: topographic map of Jebel Qattar area with mentioned sites, framed zone is expanded in Figure 7.

JQ-101 (28°01′06.31 N, 41°03′45.38 E) is located at the base of the north-eastern side of a small but distinctive cone-shaped mountain, Jebel Qattar, which reaches a maximum height of 905 m above sea level (a.s.l.). JQ-101 is in a small depression, at 810–820 m a.s.l. The site is surrounded by sand dunes to the north, east and west, with the jebel representing the southern boundary (Figure 3). Rock art is present on boulders and upper cliff faces facing JQ-101. The boulders are sometimes elaborately engraved with figures of animals such as cattle and goats and humans (some with bows and arrows). These undated representations can easily be distinguished, by both style and patination, from later Thamudic depictions, suggesting some of the rock art was produced during the Early Holocene wet phase [59], [60]. While the dating of rock art is notoriously problematic, we suggest that at least some of the rock art correlates with the occupation represented at JQ-101.

The lithic assemblage at JQ-101 occurs on, or just below the surface of homogenous orange-yellow aeolian sand sediments. The presence of lithics on the surface may be the result of deflation and erosion processes that prevented the formation of sedimentary accumulations, as is usually the case for surface sites in Southern Arabia [61]. It is also equally plausible that the presence of artefacts on the surface relates to the fact that occupations occurred on a stable sandy surface. As shown by ethnoarchaeological studies of Bedouin camps, little subsurface material remains after camps are abandoned [62]. Abandoned tent emplacements, in fact, have thin deposits less than 5 cm in thickness. As there are no signs of constructed features at JQ-101, the possibility exists that the site was occupied by nomadic peoples on a sandy surface.

### Palaeoenvironmental Studies on Nearby Lacustrine Deposits at Jebel Qattar 200

Lacustrine deposits at Jebel Qattar 200 (JQ-200) have been investigated a short distance to the southeast of JQ-101, indicating that JQ-101 was proximal to a lakeshore during the Early Holocene (Figure 3). Multiproxy analysis of the sediments at JQ-200 was conducted utilising grain size analysis, magnetic susceptibility, organic carbon and carbonate content. To determine grain size, samples of air-dried sediment were gently disaggregated in de-ionised water and analysed using a Malvern Mastersizer 2000. Mass specific, low frequency magnetic susceptibility measurements (χlf) were obtained from each sample using a Bartington MS2 meter with an MS2C sensor at 0.1 SI sensitivity [63]. Loss on ignition organic content (LOI_org_) and carbonate content (LOI_carb_) were conducted following the standard procedures described by Dean [64] and Heiri [65].

**Figure 4:**
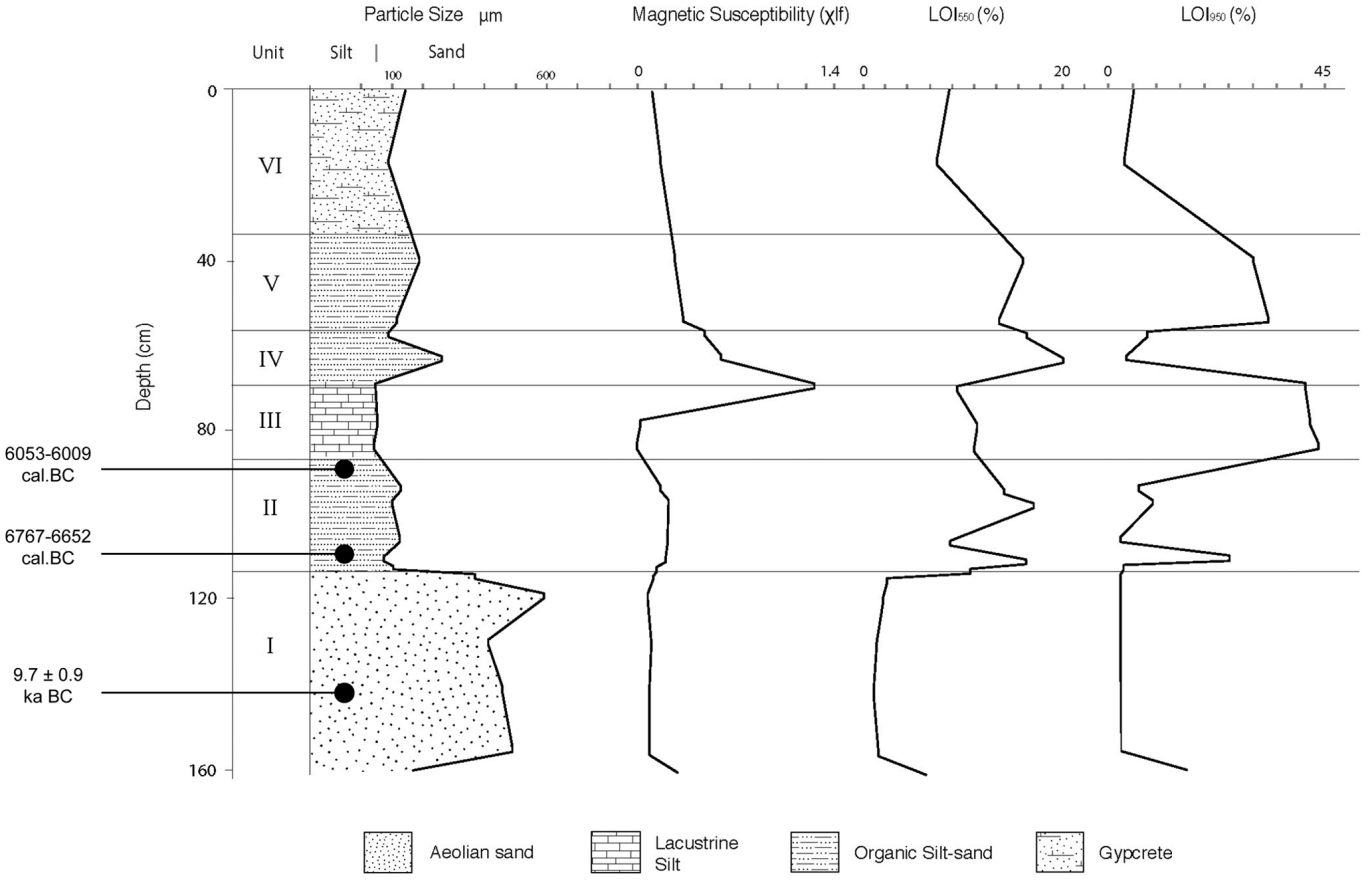
Sedimentological analyses from the palaeolake sequence at JQ-201. Showing mean particle size, magnetic susceptibility, LOI 550°C (organics) and LOI 950°C (carbonates). Radiocarbon ages are shown in cal. BC.

The section at JQ-200 comprises a 1.6 m-thick sedimentary sequence of aeolian sands, overlain by lacustrine silts and sands, which in turn are overlain by coarse aeolian sand capped by gypcrete. Grain size analysis indicates that the sedimentary sequence is comprised of two principal components that reflect lacustine and aeolian depositional processes (Figure 4).

**Figure 5:**
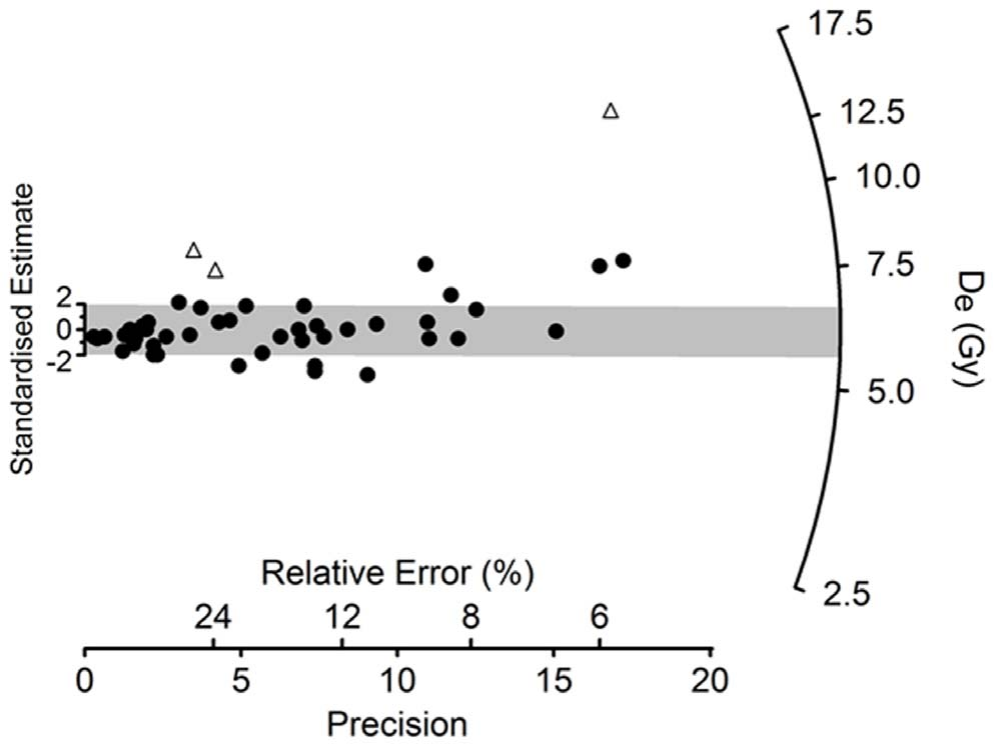
Optically Stimulated Luminescence (OSL) dating. Radial plot of the equivalent dose (D_e_) values obtained from 49 individual grains of quartz from sample JQ200-OSL1. Filled circles denote the 46 values used to calculate the weighted mean D_e_ for OSL age determination, and open triangles are the three values identified as outliers (Table 1). Each point represents the D_e_ value for a single grain. It can be read off the radial axis by extending a line from zero on the ‘standardised estimate’ axis through the point of interest, and the ‘relative error’ on this D_e_ can be read by projecting a vertical line to intersect the horizontal axis. The ‘precision’ is the reciprocal of the relative error, so the most precise D_e_ estimates lie furthest to the right. The grey band is centred on the weighted mean D_e_ estimated using the Central Age Model. Individual D_e_ values that are consistent at 2σ with this weighted mean fall within the grey band. This D_e_ distribution is overdispersed by 23±4%, which is typical for well-bleached samples of quartz that have not been disturbed since burial. See Galbraith and Roberts [71] for further explanation of statistical aspects of D_e_ estimation and display in OSL dating.

The basal Unit I (160–125 cm) comprises yellow, medium-grained, moderately well-sorted, aeolian sands, which grade into organic, sands between 125–113 cm. The granulometric values (mean particle size, skewness, kurtosis) are typical of those from active dunes within arid regions [66]. A sediment sample (JQ200-OSL1) for OSL dating was collected from a depth of 140–145 cm. Methods of sample collection, preparation and measurement (and the equipment used to make the latter) are the same as those described previously by Petraglia et al. [56], [58] for OSL dating of other sites in the vicinity. In OSL dating [67], [68], the time elapsed since a grain was last exposed to sunlight is estimated by dividing its equivalent dose (D_e_, a measure of the radiation energy absorbed by the grain during the period of burial) by the environmental dose rate (the supply of ionizing radiation to the grain per unit time over the same period). We measured 3,000 individual grains of sand-sized quartz (180–212 µm in diameter), of which most (88%) were too dim for D_e_ determination using the single-aliquot regenerative-dose procedure, and a further 10% failed a series of standard tests and checks used to reject grains with unsuitable OSL properties [69]. Forty-nine grains (1.6% of those measured) proved suitable for D_e_ determination (Table 1, Figure 5). Their D_e_ values are consistent with the grains being well bleached by sunlight at deposition and undisturbed thereafter, apart from three higher D_e_ values identified as outliers. The latter were discarded and the OSL age calculated for the remaining 46 grains from the weighted mean D_e_ (estimated using the Central Age Model [70], [71]) divided by the total dose rate. The age of 11.7±0.9 thousand years (ka) indicates that these basal sands were deposited in the terminal Pleistocene or Early Holocene. This age estimate is logically congruent with both the character and radiocarbon chronology of the overlying lacustrine sediments.

**Table 1:** Optically Stimulated Luminescence (OSL) age and supporting data for sample JQ200-OSL1.

Sample depth (cm)	Total dose rate (Gy/ka)a,b	Equivalent dose (Gy)a,c	Age (ka)^a^
140–145	0.52±0.03	6.06±0.32	11.7±0.9

a Mean ± total (1σ) uncertainty, calculated as the quadratic sum of the random and systematic errors.

b Sum of dose rate contributions from beta particles (0.113±0.006 Gy/ka), gamma rays (0.170±0.009 Gy/ka), cosmic radiation (estimated as 0.20±0.02 Gy/ka) and alpha emitters inside the quartz grains (assumed as 0.03±0.01 Gy/ka). Calculated for a water content of 5±2%, which is higher than the measured (field) water content (0.92%) to allow for the subsequent lake and swamp conditions. For each 1% increase in water content, the total dose rate decreases (and the OSL age increases) by ∼1%. The beta and gamma dose rates were measured by low-level beta counting and field gamma spectrometry, respectively, using the same equipment and procedures as described by Petraglia et al. [56], [58]. The cosmic-ray dose rate was calculated following Prescott and Hutton [125], taking into consideration the time and rate of deposition of the overlying lacustrine and aeolian units.

c 3000 grains were measured and analysed as follows: preheat of 260°C for 10 s (natural and regenerative doses) or 220°C for 5 s (test doses); green laser stimulation at 90% power for 2 s at 125°C; OSL signal from initial 0.2 s and background from final 0.3 s of stimulation; use of saturating exponential function to fit dose-response curves and estimate the equivalent dose (D_e_). The D_e_ measurement uncertainty includes variation in background-corrected OSL counts, laser repositioning error (2% per measurement) and curve-fitting errors estimated by Monte Carlo simulation. Forty-nine grains (1.6%) satisfied all of the criteria used to identify grains with suitable OSL properties for reliable D_e_ determination [68]. Of the 2951 grains rejected, 2640 were discarded because the OSL signals were too dim to discern above background, and 311 were rejected on the basis of feldspar contamination, high ‘recuperation’ or a poor ‘recycling ratio’. The weighted mean D_e_ was calculated for the remaining grains using the Central Age Model [70], after discarding three values (Figure 5) that differ from the weighted mean by >2σ and have normalised median absolute deviations of >1.5 [126]. Including these outliers increases the weighted mean by 0.38 Gy, which is within statistical error of the tabulated D_e_ value. The total uncertainty on the weighted mean includes the additional spread (‘overdispersion’) of 23±4% in D_e_ values beyond that associated with measurement uncertainties for individual grains [71] and a relative error of 2% for possible bias in the calibration of the laboratory beta source.

A distinct black layer was observed at a depth of 114 cm. From 113 to 88 cm there is a distinct change to blue/grey banded silts and fine sands, which are poorly sorted (Unit II). The organic content increases to 7–15%. Carbonate content is low in the darker banded organic sediments and increases up to 22% in the lighter banded marls. Several layers contained charcoal and reed stem fragments. Mollusca and plant macrofossils were also observed. Two radiocarbon (^14^C) samples were measured on charred seeds (Cyperaceae and *Carex* sp.) and charcoal fragments at the SUERC AMS facility in East Kilbride, Scotland, and a sediment sample was collected from the site for optically stimulated luminescence (OSL) dating (Table 2), from depths of 112–113 cm (6,642–6,978 cal. BC or 8,591–8,927 cal. BP) and 90–92 cm (5,990–6,069 cal. BC or 7,939–8,018 cal. BP). Calibrated radiocarbon ages are expressed in calendar years and, hence, can be placed on the same timescale as the OSL age.

**Table 2:** Conventional and calibrated radiocarbon ages for site JQ-200.

Sample depth (cm)	Sample lab code	Conventional ^14^C age (BP)^a^	Dated material	δ^13^C (‰)	Calibrated ^14^C age ranges(cal. BC and cal. BP)^b^
90–92	GU27206	7,160±30	Charred reed stem fragments, Cyperaceae and *Carex sp*. seeds	−25.0	6,009–6,052 cal. BC (68% CI)5,990–6,069 cal. BC (95% CI)7,958–8,001 cal. BP (68% CI)7,939–8,018 cal. BP (95% CI)
112–113	GU27207	7,880±30	Charred reed stem fragments	−25.0	6,652–6,766 cal. BC (68% CI)6,642–6,978 cal. BC (95% CI)8,601–8,715 cal. BP (68% CI)8,591–8,927 cal. BP (95% CI)

a Radiocarbon years before present (BP).

b Calendar years BC and BP, calibrated using the IntCal09 data set [127]. Age ranges are listed at both the 68% and 95% confidence interval (CI).

The third main unit comprises cream-colored carbonate-rich (∼40%), poorly sorted, lake silts between 88 and 65 cm (Unit III). This unit is characterized by low magnetic values, which reflect the diamagnetic qualities of carbonate-rich material. A 10 cm band of organic-rich fine-medium poorly sorted sand overlies these silts. There is a noticeable peak in mean particle size from 100 to 300 microns between 65 and 55 cm. Between 68 and 55 cm (Unit IV) there is a marked increase and peak in magnetic susceptibility. Cyperaceae seeds were noted at 61 cm. This would suggest a lowering in lake level and an associated increase in sediment particle input. From 55 to 40 cm, cream-colored, carbonate-rich sediments are present, indicating a rise in water table (Unit V). The particle size range is coarser that the previous cream silt-rich band. The uppermost unit (40 cm to the surface) comprises medium sands capped with a fairly massive gypcrete (Unit VI).

## Field Methods and the Lithic Assemblage

### Excavation and Collection Procedures

**Figure 6:**
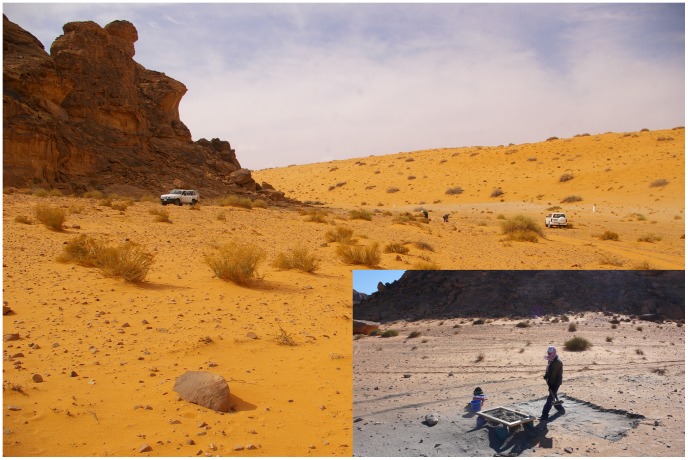
General views of JQ-101 site while excavated and surveyed.

Archaeological field work at JQ-101 was composed of surface collections and trench excavations. A random surface collection was made across the site, including the collection of selected tools (retouched blanks), technical pieces (rejuvenation flakes, burin spalls), blades and cores. Each of the collected items was recorded with a GPS location. Excavations were comprised of six 2×3 m trenches placed across the site - two at the western part of the site, three to the east and one in the centre - to examine areas with high to low density artefact scatters and to determine whether subsurface deposits were present (Figure 6). All excavated sediment was passed through 5 mm mesh sieves to recover small artefacts and to check for the presence of faunal remains and non-lithic artefacts (neither were identified).

### Raw Materials and Assemblage Composition

**Figure 7:**
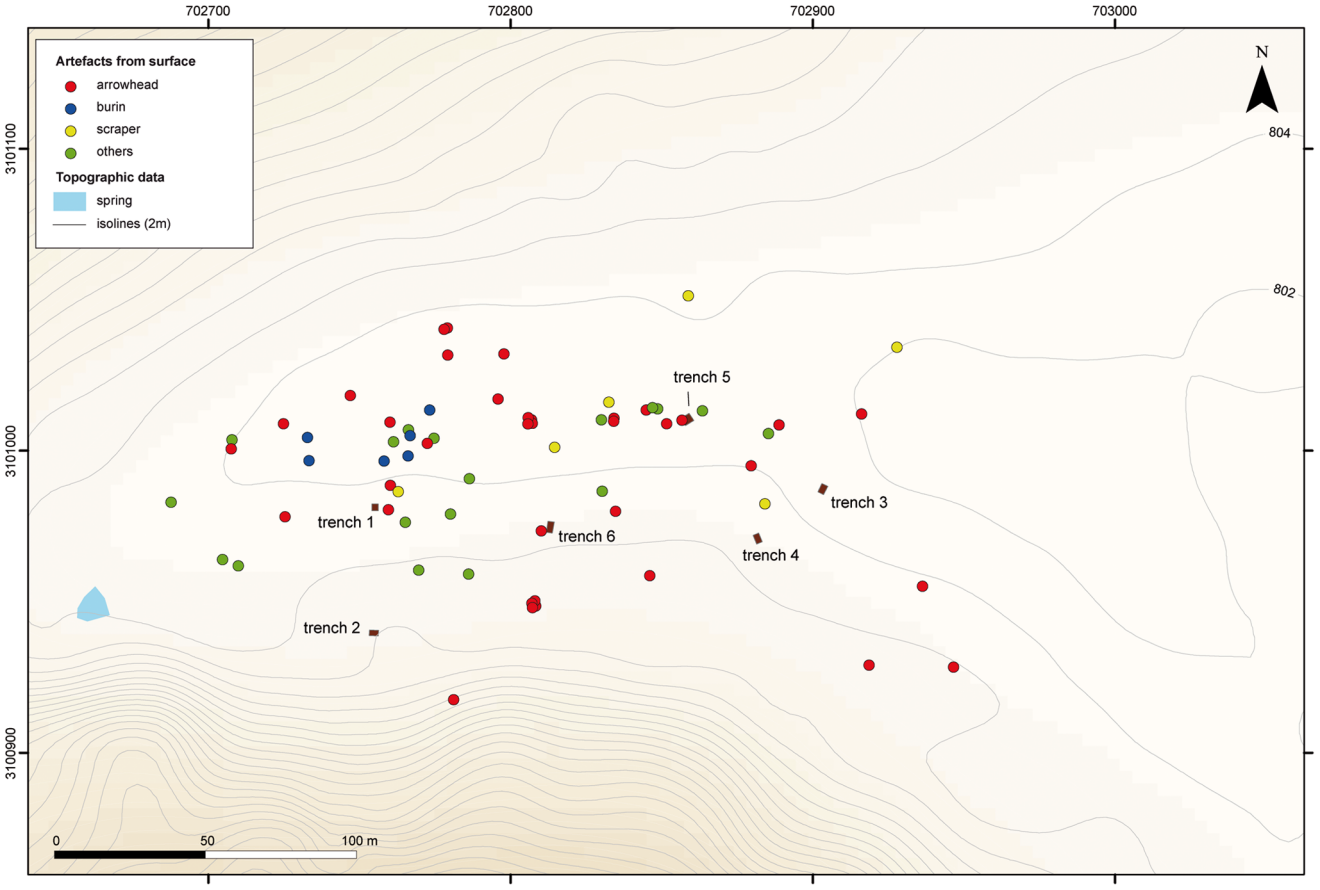
Map of artefacts distribution on surface at JQ-101, with trenches localisation.

A total of 887 lithics was collected; 73 from the general surface and 814 from the excavation units surface or sub-surface (Table 3). Chipped stone tool artefacts were the exclusive finds identified, and no groundstone or faunal remains were recovered. As each of the surface tools was geo-referenced, spatial distribution plotting of the artefacts shows areas with slightly higher diagnostic finds (Figure 7).

**Table 3:** Total number of lithics from the general surface collection and from the six trenches (systematic collection).

Location	tools	non-tools	TOTAL
JQ-101 surface	67	6	73
JQ-101 TRENCH 1	6	441	447
JQ-101 TRENCH 2	0	66	66
JQ-101 TRENCH 3	5	60	65
JQ-101 TRENCH 4	0	12	12
JQ-101 TRENCH 5	14	83	97
JQ-101 TRENCH 6	4	123	127
**TOTAL**	**96**	**791**	**887**

Prior to the excavation of the six trenches, all surface materials within their boundaries were collected, including undiagnostic flakes, chunks and micro-debris (>5 mm). Each of the six trench excavations recovered shallowly buried lithic artefacts, the majority derived from the first five centimetres. The trenches produced variable counts of artefacts, ranging from a low of 12 to a high of 447. Subsurface testing extending to a depth of up to two meters was conducted for each trench, revealing only culturally sterile sand. Trench 1 was excavated to a depth of three meters, but with the same negative archaeological result.

The 887 artefacts collected from the surface and from the excavations were mainly composed of flakes and chunks (Table 4). The flakes were generally small. Retouched tools comprised projectile points, burins, scrapers and borers. Among the 96 retouched tools, projectile points are the most numerous (n = 65), followed by rarer tools, including seven retouched blades, one retouched flake, six scrapers and four tanged scrapers, nine burins, two geometrics and one *pièce esquillée*.

**Table 4:** Artefact types (tools, cores and technical pieces).

TYPES	Number
undiagnostic arrowheads	55
El-Khiam points	6
Helwan points	4
burins	9
retouched blades	7
scrapers	6
tanged scrapers	4
blades	2
burin spall	2
cores	2
geometrics	2
rejuvenation flake	2
bifacial piece	1
pièce esquillée	1
retouched flake	1
TOTAL	104

The raw materials are comprised of mainly good quality chert (50.9%) and quartz (49.1%), almost in equal proportion (Table 5).

**Table 5:** Raw material types (counts) from the six trenches (systematic collection).

Location	chert	quartz	TOTAL
JQ-101 TRENCH 1	199	248	447
JQ-101 TRENCH 2	32	34	66
JQ-101 TRENCH 3	45	20	65
JQ-101 TRENCH 4	10	2	12
JQ-101 TRENCH 5	71	26	97
JQ-101 TRENCH 6	57	70	127
**TOTAL**	**414**	**400**	**814**

Flakes were produced from different types and textures of chert, displaying a wide range of colours. The cherts were generally good quality siliceous materials, suitable for fine retouching using pressure flaking. The surface distribution of these materials was distinctive, suggesting that material concentrations were present. Small cores of chert were made from pebbles, likely procured from an unknown palaeoriver bed. At present, no chert outcrops have been identified in the vicinity of Jubbah. Interestingly, the most ubiquitous raw material, ferruginous quartzite, was ignored by the JQ-101 inhabitants, though this material was commonly used by Middle Palaeolithic populations on the opposite side of Jebel Qattar [56], [58].

The white quartz is a locally available raw material, found in the form of small nodules or pebbles. The clasts occur in the sandstone bedrock and as part of gravel bar spreads in the vicinity of Jebel Qattar. The quartz cores, flakes and chunks appear to be the result of bipolar percussion, most probably on an anvil. This method of reduction produced irregular and non-standardized blanks. None of the quartz artefacts are particularly culturally diagnostic. Notably, crystal quartz was present, although only two tools of this material were recovered.

There appears to be some horizontal patterning of the artefact assemblage based upon the concentration of diagnostic pieces and raw materials on the surface and the high density of artefacts in certain zones of the site on both the surface and in excavated contexts. It is possible that these site-wide patterns reflect different activity areas across the site or different phases of occupation.

### El-Khiam Points

**Figure 8:**
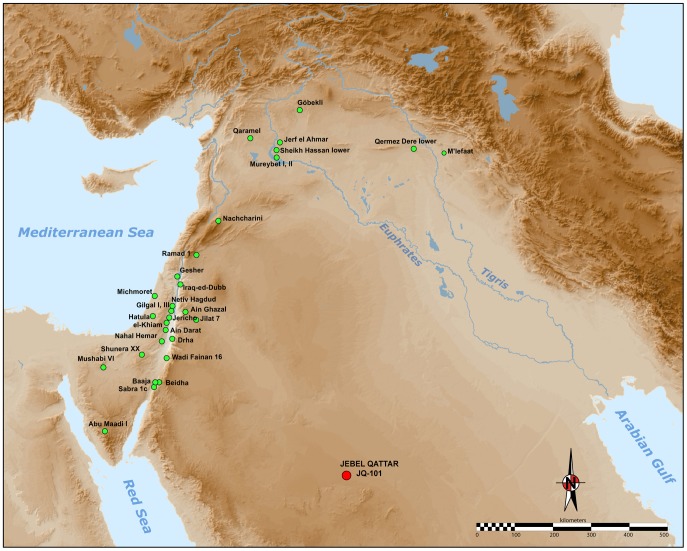
Map of the Levantine sites with El Khiam points. It is based on maps in Kozlowski and Aurenche [73]. JQ-101 lays more than 500 km from the “core area”.

A total of six arrowheads from JQ-101 can be classified as El-Khiam pointtypes. This type is well known from the Levant [42], [72], [73] and is dated to the PPNA in the Northern Levant, but can be extended to the beginning of the PPNB (Early PPNB) in the Southern Levant (Figure 8).

Originally discovered near Wadi Haritoun in the Judean desert on the shore of the Dead Sea, the El-Khiam site was dated to the Epipalaeolithic and the Early Neolithic [74]. This site gave its name to a culture called the Khiamian (c. 10,000-9,500 cal. BC; [75]), which is particularly characterized by the El-Khiam points [42], [72], [76]. The El-Khiam points appear in a Natufian (Epipalaeolithic) context from the Sinai (e.g. Abu Madi site: [77]), to the Jazira region in Northern Syria, passing through the Levant (e.g. El-Khiam site, Lebanon: [78]; Gesher site, Israel: [79]), and the Syrian Middle Euphrates (e.g. Mureybet site; [80]). They are classically made on small regular bladelets and are characterized by a concave or rectilinear base and by parallel bilateral notches obtained by abrupt retouch that were used for hafting [81]. The pointed part is often ventrally retouched on one or both convergent edges.

The El-Khiam points replaced the geometric microliths and the Harif points that, until then, were used to arm arrows. They are present in the ancient phase of the Nemrikian and are persistent in the Mureybetian, where they are gradually replaced by the Helwan points [82]. In the Levant, this point type is present between about 10,500 and 8,500 cal. BC. The El-Khiam point is the *fossile directeur* of the Khiamian industries (associated facies are Classical Khiamian, Sultanian, Qermezian) and is, more broadly, considered as a major *fossile directeur* of the entire PPNA. El-Khiam points from South Levant are usually thicker than in other parts of its known distribution and are more recent (Early PPNB), but with a persistent PPNA tradition (F. Abbès pers. comm. 2012).

**Figure 9:**
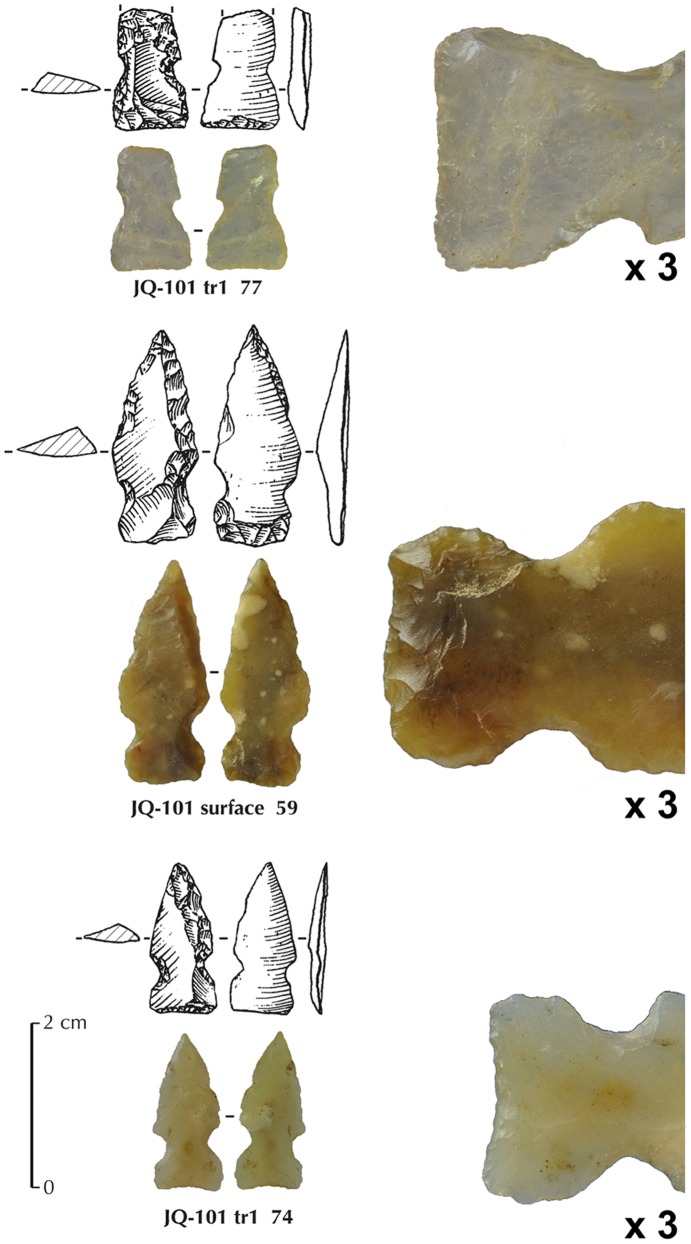
Three El-Khiam points from JQ-101. The enlarged views represent the basal ventrally retouched parts (x3). Top one (broken) is in crystal quartz.

El-Khiam points from JQ-101 (Figure 9) show the classical concave or linear base, manufactured by inverse or direct retouch. The point is shaped by inverse or direct retouch along one or two edges. The notches are produced by direct pressure. General dimension ranges for complete pieces are 18–25 mm in length, 8–10 mm in width and 2–3 mm in thickness. At JQ-101, regular bladelets do not occur in the debitage, and reduction of naviform cores and small unidirectional cores are absent. Rather, flake production seems to be less organized, with the use of regular flakes for arrowhead blanks. The use of plain flakes takes advantage of the natural cutting edges of the flake and the non-predetermined shape of the blank. The knappers shaped the point, attempting to reproduce the ‘classic’ forms of El-Khiam points, which involves using more retouch than is usual in the Levant. A Levantine influence can be observed in the shaping of the final point form. This technique does not involve technical competence to produce blanks such as bladelets.

### Helwan Points

**Figure 10:**
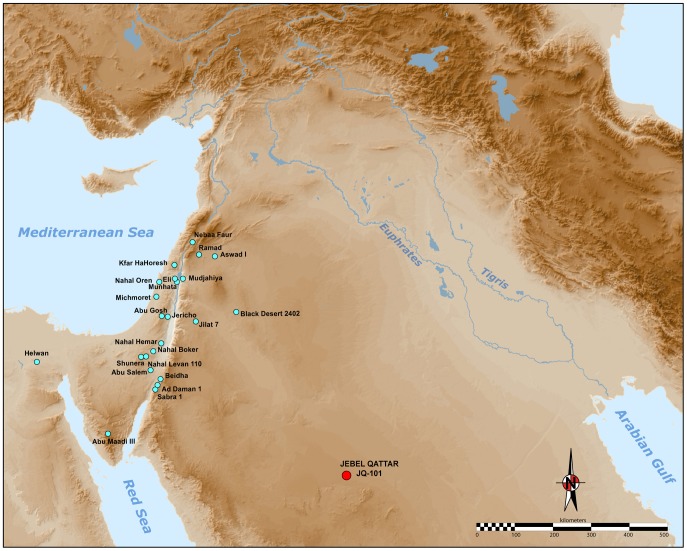
Map of the Levantine sites with Helwan points. It shows the Abu Salem points sub-type, based on maps in Kozlowski and Aurenche [73]. JQ-101 lays more than 500 km from the “core area”.

A total of four Helwan points were recovered from JQ-101. This type of point was first documented in Egypt, in the governorate of Faiyum, 25 km south of Cairo, where they were accompanied by a range of microlithic and blade tools. ‘Helwan Points’ described by Caton-Thompson and de Morgan [83] consisted of retouched edges, with distinctive notches and tangs. Similarities between these point types and those found in Syria and Palestine are apparent (Figure 10). Gopher [83] suggests a spread of the Helwan point tradition from the Middle Euphrates to the rest of the Levant. According to Aurenche and Kozlowski [7], Helwan points are characterized by a short tang that replaces the concave base of the El-Khiam points and are considered by the authors as a ‘logical’ development of the El-Khiam points [7].

Helwan points are known from the Highlands of Northern Syria to Egypt and are represented in the Mureybetian, the Aswadian, and the Early PPNB of the southern Levant. They are persistent in the early phase of the Big Arrowhead Industries (BAI), perhaps representing the embryo from which the tang developed [7]. Aurenche and Kozlowski [82] differentiate three sub-types within the Helwan tradition: Sheikh Hassan points, Aswad points and Abu Salem points. This last sub-type is very comparable to the arrowheads found at JQ-101, and more precisely to the Abu Salem *small type*. Its geographical distribution is clearly related to the southern Levant [73], between Ramad and Beidha through the Abu Salem site in the Sinai and Jilat 7 to the east. In terms of dating, the Helwan point is found in the Levant between about 9,500 and 8,000 cal. BC. The Abu Salem sub-type is the latest of the Helwan points in the Levant, with the earlier examples dating to about 8,000 cal. BC. They are present at Ramad around 7,000 BC cal. and might persist in the southern Levant until the end of the PPNB, in places such as Jericho, Munhata and Nahal Oren [82]. In Jordan, the most recently dated examples date to 7,600 cal. BC, disappearing at 6,800 cal. BC at the site of Beidha [84]. However, despite the presence of Helwan points in the southern Levant, the mechanism by which they could have spread to Egypt is unclear. This type now seems to be more common in the Levant, rather than Northeast Africa, with many occurrences in Syria, Lebanon, Jordan and Israel.

**Figure 11:**
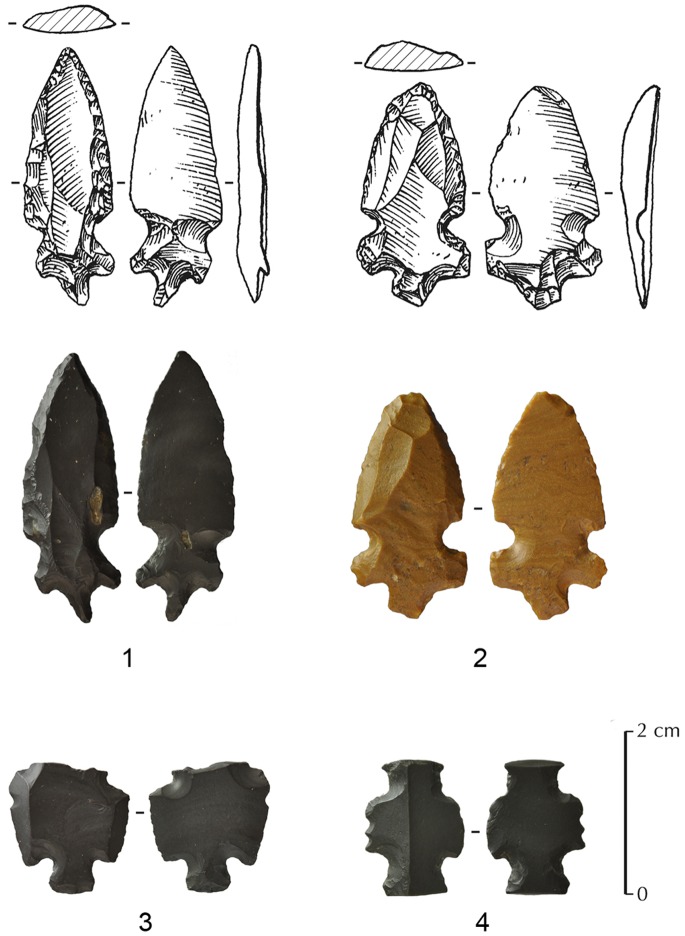
JQ-101 Helwan points (Abu Salem points sub-type). 1,2 are complete; 3 is fragmentary; and a possible Gilgal truncation (4).

Helwan points from JQ-101 (Figure 11) are metrically variable. They have a short tang, in the direct continuation of the two lateral notches mostly made by inverse single retouch. The point is shaped by direct retouch along the two edges from the upper part of the notches. General dimensions for complete pieces range from 27 to 32 mm in length, 12–14 mm in width and 3–4 mm in thickness. As observed on the JQ-101 El-Khiam points, the production of the blank for Helwan points appears not to be well-standardized. Bladelet reduction again appears to be absent. Despite the recovery of only four Helwan points at JQ-101, it is possible to demonstrate that the blanks were chosen for their natural flake properties, which were not predetermined shapes. Helwan points at JQ-101 nonetheless mark an unusual type, never observed before in this region.

### Tanged Points

**Figure 12:**
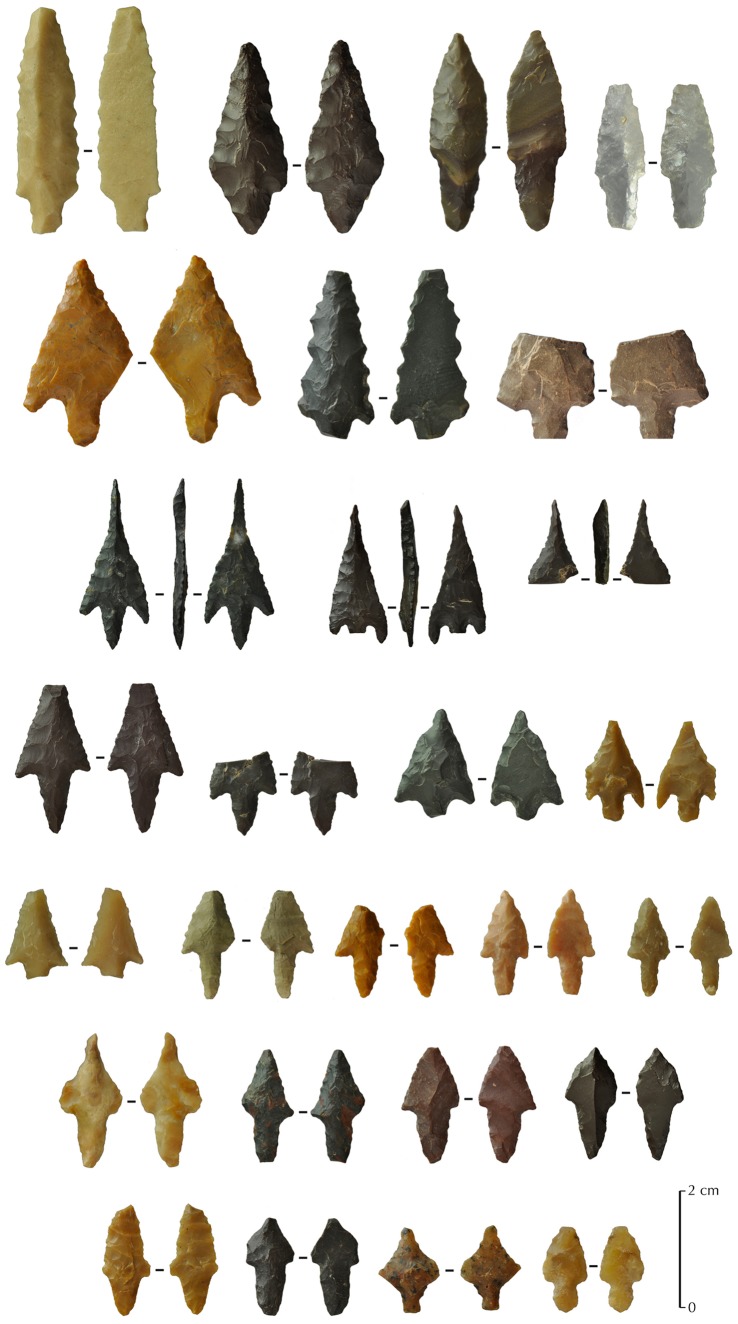
Others types of projectile points from JQ-101. Examples of different types of tanged with no barbs or barbed and tanged arrowheads.

Other types of projectile points were found at JQ-101. They are of two main types: tanged with no barbs or barbed and tanged arrowheads, with a total number of 55 artefacts, complete or fragmentary. They are all bifacially shaped by pressure retouch and most of them have a symmetrical profile (Figure 12).

In comparison with the Levantine types, tanged arrowheads and arrowheads with lateral barbs and tangs at JQ-101 are more typical of the Late Neolithic (PN period), and even the Chalcolithic [85]. Garrard et al. [53] noted typologically related Chalcolithic sites elsewhere at Jubbah, but still not clearly dated, so it is possible that JQ-101 was reused at this time. Temporary lake formation, or at least the presence of marshy conditions, may have occurred repeatedly in the Holocene and attracted human groups. Some of the lithic types found at JQ-101 are known from other sites in southern Levant, such as the ha-Parsa point, the Nizzanim point and the Herziliya point, all dated to the PN period [42].

The possible younger age of these points may be suggested by their variant morphologies and different types of pressure retouch scars on some points. Some of the finest points show extremely delicate retouching of tang, barbs and pointed part, prompting suggestions that a copper pointed tool may have been used in pressure retouching of some arrowheads (P. Bodu pers. comm. 2012). Such a tool would be too recent to be compatible with the PPN period.

The tanged projectile points from JQ-101 can also be compared to southern occurrences in the Arabian Peninsula, notably known tanged types that date to the 5^th^ millennium BC [86]. However, other occurrences of the common bifacial barbed and tanged arrowhead types are also known from much earlier sites, notably in Yemen, dated around 6,500 cal. BC (Khuzmum site or HDOR-538 and HDOR-561 sites: [61]). Some of the points at JQ-101 might even relate to some types known in Southern Arabia, such as trihedral points [61], [87], [88]. Typical examples of Yemeni or Omani classical trihedral points are nevertheless not entirely convincing at JQ-101, as the retouch type and sizes are different.

In sum, these observations and comparisons of projectile points highlight several key issues. The lithic assemblages from JQ-101 include the use of El-Khiam and Helwan points, two classic types known from the Levantine Neolithic and never described before in the Arabian Peninsula. At least two of the JQ-101 points were made on bladelets, further indicating a possible cultural affiliation with Levantine PPN technology. The variation in projectile points at JQ-101 indicates that the site was occupied in different phases of the Neolithic. The Early Holocene lake would have attracted game and mobile hunting groups who likely employed bows and arrows to pursue game. The presence of bifacial tanged arrowheads, with and without barbs, is related to common types in the Levant and Southern Arabia, but for a broad period of time from the Neolithic to the Chalcolithic. These types need to be clearly dated at JQ-101 in order to demonstrate a techno-temporal relationship. Their typological aspects cannot be solely taken into account for a clear chronological attribution. As a fairly sheltered spot, next to a spring and a lake, it is likely that JQ-101 was visited by groupsat numerous times.

### Other Tools

#### Burins

**Figure 13:**
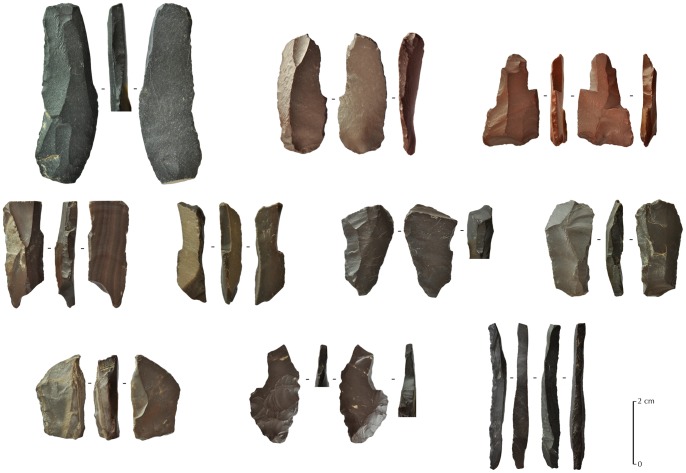
Burins from JQ-101. One long burin spall is shown bottom right.

A total of nine burins (Figure 13) and three burin spalls were collected. Eight are simple burins from the distal part of a blade or an elongated flake, made on a plain surface or on a truncation. One example is a double burin. This kind of tool is unknown in the toolkits of Neolithic Arabia, and can be, again, putatively linked with the Levantine Neolithic, where many types of burins are common in the PPNA and PPNB [89]–[93].

#### Scrapers

**Figure 14:**
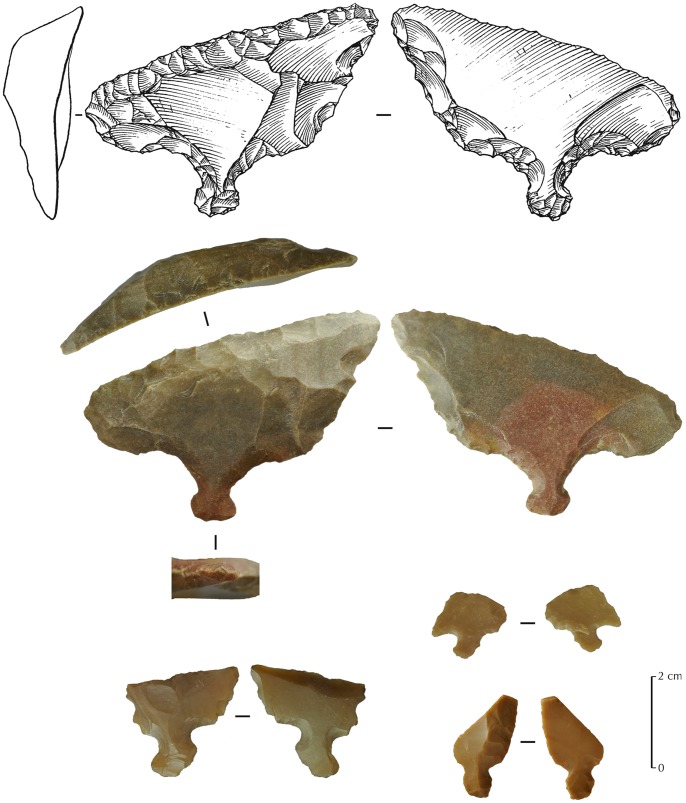
Tanged scrapers from JQ-101. The upper one (drawing and photography) is complete, while the three others are fragmentary.

A total of ten scrapers were recovered. Six are made on undiagnostic flakes. A few examples of thumbnail scrapers were made on flakes. Such end-scrapers on flakes are known from both from the Levant and from Arabia in the Early Holocene. However, four of the scrapers have a tang, which is a peculiarity never described before in Arabia (Figure 14). Such a type of scraper is also unknown in the southern Levant, but is somewhat reminiscent of artefacts categorized as regular scrapers, notably at Byblos in Lebanon [41] and at Fara in northern Egypt [94]. An example from JQ-101 is a scraper made on a thick flake, with an active edge along the side, and a small bifacially shaped tang at the opposite side.

#### Retouched blades

A small number of bladelets and small blades are present (nine were collected), though no laminar or naviform cores were recovered. These elements are often retouched (seven retouched bladelets), mostly by direct and non-invasive pressure retouch. Two geometric microliths were also recovered and one possible truncated and notched piece, resembling the Netiv Hagdud truncated tools from the PPNA forms of the southern Levant [95]. These latter artefact types are another good indicator of a PPNA/Early PPNB occupation. Another interesting artefact attributable to the Early PPNB period in the southern Levant is a probable Gilgal truncation [96], dated to the 10^th^–9^th^ millennium cal. BC and known from the southern Levant to the Syrian Jezirah. While acknowledging the limitations of a sample size of one, we note the clear similarity between the JQ-101 and Levantine artefacts (Figure 11∶4).

## Discussion: implications of a PPNA/PPNB presence in Northern Arabia in the wider Eastern Mediterranean context

### Pre-Pottery Neolithic Dating in the Levant and its Potential Application to Saudi Arabia

The discovery of typical PPN arrowhead types in the south of the Nefud Desert of Saudi Arabia marks an important step in the understanding of the dispersal of Neolithic people in arid regions neighbouring the Fertile Crescent. JQ-101 is a key site, with culturally diagnostic points in association with a chronometrically dated palaeolake, which can be presumed to correspond with the human occupation. The discovery indicates that the arid regions of southwest Asia contain important information on the cultural and demographic changes of the early Holocene. The archaeology of the arid areas of this zone will likely be rather different in character compared to the more fertile, and more intensively researched areas, of Southwest Asia. A robust understanding of entities such as the PPN necessitates understanding both core and periphery areas and interactions between populations.

Accurate dating for a superficially technologically homogenous period such as the PPNA or the PPNB is somewhat problematic, as major differences occur in different regions of the Levant and the greater region of the Fertile Crescent. Moreover, owing, in part, to the variable quality of work by numerous international teams over the last sixty years, chronological schemes are often debated. Nevertheless, the ASPRO (*Atlas des Sites du Proche-Orient*: Atlas of Near East archaeological sites) chronology is an attempt to outline a nine period dating system of the ancient Near East used by the Maison de l’Orient et de la Méditerranée (CNRS Lyon, France) for archaeological sites aged between 14,000 and 5,700 BP [30]. Despite new excavations, discoveries, and dates, the ASPROchronological framework is still in use as it has not been falsified by additionaldiscoveries.

Direct comparisons with the Levantine Neolithic are also complicated by the nature of the JQ-101 site, which was most probably a temporary campsite and, therefore, indicative of a more nomadic lifestyle compared to the classical sedentary sites in the Levant. The correlation of lithic types between north Arabia and the Mediterranean Levant are, however, evidence for connections, adding a dating hint to at least some of the earliest Holocene occupations at Jebel Qattar.

Thus, it is clearly possible to associate the El-Khiam and Helwan points to the PPNA and the early PPNB, between 10,500 and 8,000 cal. BC. Other occupations can be dated to later periods, between 7,000 and 5,000 cal. BC. Chronological association for other projectile point types (the tanged points) is more problematic, as they occur across a wider temporal and cultural spectrum. Their presence indicates the repeated use of the site over centuries and millennia.

### Palaeoclimatic Evidence

The sedimentary sequence at JQ-200 demonstrates that a water body existed during the Early Holocene beneath the slopes of Jebel Qattar. The presence of PPN lithics at JQ-101 denotes occupation along a lakeshore during the Early Holocene. The OSL age of 11.7±0.9 ka (9.7±0.9 ka BC) for the base of the sequence (Figure 4) indicates that the aeolian sands underlying the lacustrine sediments were deposited during the terminal Pleistocene or Early Holocene, which is consistent with reports of Late Glacial dune activity in other regions of Arabia [97], [98].

At Tayma, on the western edge of the Nefud, a perennial lake with a minimum depth of 13 m and a surface of 18.45 km^2^ existed during the Early Holocene from c. 8,050 cal. BC (c. 10,000 cal. BP). Palaeo-rainfall during the Early Holocene was at least 300% greater than today, with a calculated minimum annual precipitation of 150±25 mm [99]. In the Nefud Urayq, lacustrine sediments were dated to c. 7,450 cal. BC (c. 9,400 cal. BP) [100], [101]. Further southwest, in the Rub’ al-Khali, the onset of lacustrine conditions occurred at 7,850–7,650 cal. BC (c. 9,800–9,600 cal. BP) at Mundafan [57], [102], [103]. Figure 2 shows that the palaeolakes that have been studied in the region so far are just a small subsample of those that are actually present. Thus it appears that in humid phases, lakeshore settings for sites such as JQ-101 would have been commonplace.

At JQ-101, the onset of wet conditions during the Holocene is interpolated to have occurred at c. 8,050 cal. BC (c. 10,000 cal. BP). Lacustrine conditions developed during the Early Holocene with several fluctuations in water depth as denoted by alternating phases of deeper water lacustrine marls and organic silts reflecting shallower water swampy conditions. These beds could be traced across the site in several exposures. Similar sedimentary conditions have been reported from interdune depressions elsewhere in the Nefud region [101]. The presence of charred reed stem fragments and seeds of Cyperaceae and *Carex* species at JQ-200, supports the notion of marshy conditions at the site. At Jubbah, around 13 km west of Jebel Qattar, Garrard et al. [53] dated a palaeosol to c. 5,600 cal. BC (c. 7,550 cal. BP).

Information on the Holocene palaeoclimate from the northern section of the Arabian Peninsula is restricted to interdunal sediment records from the northwestern branch of the Nefud erg which, according to investigations by Whitney et al. [100] and Schulz and Whitney [101], provide evidence for the presence of shallow lakes and swamps c. 7,550–5,850 cal. BC (c. 9,500–7,800 cal. BP). Signs of Early to Mid-Holocene pedogenesis, implying wetter conditions, were found nearby at Jubbah [53]. The analysis of pollen and plant remains from the Nefud by Schulz and Whitney [101] led them to conclude that the Early Holocene environment was characterized by semi desert or savannah-like conditions.

Given the location of the JQ-101 lithic assemblage, Early Holocene conditions must have been wet enough to provide both freshwater resources and sufficient vegetation cover and faunal populations within the landscape for humans to migrate into and exploit the region around the Jubbah basin. The source of Early Holocene moisture across the Nefud region is not yet fully known. A northward shift of the intertropical convergence zone (ITCZ) and associated monsoonal precipitation during the Early Holocene is generally accepted as the main source of precipitation during the Early Holocene in the south and southeast of the Arabian Peninsula [104]–[106]. From the analysis of sediment cores collected from the northernmost Red Sea, Arz et al. [107] concluded that monsoonal rains did not reach northern Arabia during the Early to Mid-Holocene. They suggested that the source of moisture was derived from Mediterranean Westerly air masses. Therefore, there is a strong possibility that similar systems were responsible for the Early Holocene rainfall across the Levant and into the Nefud.

### Evidence for a Levantine Neolithic Incursion into Arabia: How Strong?

The absence of direct radiometric dating of surface artefacts from JQ-101 is tempered by the indisputably Levantine projectile point types and the absolute dates on the neighbouring palaeolake (JQ-200). The presence of these distinctive Levantine style points is a geographical extension to the south, the first time that they have been identified in the Nefud Desert of Saudi Arabia.

The El-Khiam and Helwan points at JQ-101 either represent: (1) a physical movement of people by population dispersal, or (2) a cultural diffusion or influence.

The independent, convergent development of stylistically diagnostic PPN points in northern Arabia is unlikely, as the idiosyncratic morphology and dimensions of the point forms are very similar to those from the Levant. That said, while the end products are similar the *chaîne opératoire* is somewhat different. Firstly, the production of blanks is not clearly oriented to regular bladelet production and there is no evidence of bidirectional flaking of cores. Secondly, the shaping of the tool itself, by pressure retouch, seems much more invasive than with the classical El-Khiam points. These technological differences in both blank production and retouch therefore suggest that a wholesale physical movement of populations is less likely, and a stylistic influence on local populations is more probable, perhaps introduced by small numbers of individuals. Regardless of interpretation, a clear geographic and stylistic link with the Levant is present at JQ-101, while no evident link can be made with South Arabia, whether from southern Saudi Arabia [57], Yemen [61], [88], the Oman Peninsula [108], or the Arabian Gulf [109]. The JQ-101 discovery therefore adds a new dimension to discussions of the development of the Arabian Neolithic [110], where interpretations have been divided between those favouring a dispersal of Neolithic people into Arabia and those who emphasise indigenous developments in southern Arabia [111], [112].

In terms of cultural links and movements, two scenarios can be conceived, perhaps operating in parallel: (1) migration of limited numbers of social groups or members of groups from not too distant localities for special purposes (hunting, ritual, exploration); (2) more distant incursions with acculturation of local groups. The second scenario is implied at the Acila site 36, in Qatar [113]–[115], where PPNB-like points (Amuq or Byblos type) and bidirectional (naviform?) cores have been recognized. These cultural styles seem to have disappeared in Qatar as quickly as they appeared and apparently suggest a short-term incursion of groups with no long-term impact on local traditions [116]. The Acila example is the only example of its kind in the Arabian Gulf region and JQ-101 represents the only other comparable occurrence of PPN technology south of the Levant.

The evidence for the movement of some people and the diffusion of ideas into the JQ-101 and Acila site regions contrasts with archaeological information in southern Arabia, which highlights population continuity from the Pleistocene into the Holocene. In both cases, it appears that indigenous populations were present across Arabia, indicating that limited population introductions and cultural influences took place in the context of an occupied landscape [117], [118]. Thus, the hypothesis of a *tabula rasa* or empty landscape seems to have been firmly falsified, at least for the last glacial cycle.

The existence of refugial areas in Arabia has been postulated for some time [117], [118], yet, there has been limited archaeological evidence to support such hypotheses. Fedele [119] reports ‘pre-Neolithic’ archaeological evidence dating to the early Holocene in the highlands of Yemen. In Dhofar, Oman, the sites of Al-Hatab, Ghazal, and Khamseen date to c. 14-7 ka and contain a distinctive form of lithic technology with an indigenous character [120]. The discovery of these sites in Dhofar challenges the notion that the Holocene peopling of Arabia, associated with lithic types including Fasad points reflects, perhaps exclusively, the movement of Neolithic people from the north [121]. While much remains to be discovered, it does appear that in at least southern Arabia indigenous populations were present at the time the Neolithic was developing to the north. The spatial and temporal limits of these southern Arabian populations are at present unclear.

Complementing such archaeological discoveries are a number of recent genetic studies, e.g. [112]. While recognising the caveats of genetic evidence, such as on-going debates about mutation rates, the current evidence seems to support the archaeological picture discussed above. Great care needs to be taken when interpreting genetic data, particular inferring the location of populations tens of thousands of years ago from where populations are found today. Nevertheless, the existence of basal haplogroup N clades in southern Arabia has been interpreted as indicating population continuity in southern Arabia from broadly the middle of Upper Pleistocene [122]. Other haplogroups, including R0a, indicate divergences to produce variants found in southern Arabia in the terminal Pleistocene and early Holocene [123]. Other haplogroups seem to represent both dispersals from north of Arabia associated with the movement of ‘Neolithic people’, and more recent historical processes such as slavery (see, for instance, references in Groucutt and Petraglia [111]). The combination of archaeological and genetic data thus indicates that early and mid Holocene Arabia saw both the movements of ‘indigenous’ people and the arrival of a limited number of new people and ideas from the north. The relative degree to which these demographic and cultural exchanges occurred across Arabia remains a central research topic. The discovery of JQ-101 hints at of some of the complexity of demographic processes occurring in early Holocene Arabia.

## Conclusions

The new discoveries reported herein, in an archaeologically poorly-known region of the world, contribute to our understanding of the relationship between the Levantine Neolithic core region and its peripheries. The role of population histories in the desert zones bordering the Fertile Crescent needs to be explored in much more detail in the future. The JQ-101 site, located in the Nefud Desert, provides an important point of reference for understanding connections with terminal Pleistocene and early Holocene groups of Mediterranean Southwest Asia and autochthonous culture(s) of southern Arabia at the same time.

At JQ-101, the recovery of distinctive stone tool styles are examples of a PPN influence from the Levantine core area to the southern Nefud Desert interior, at an approximate distance of 500 km. The recent identification of JQ-101 shows the great potential and high reward of further Neolithic research in Saudi Arabia, especially in the desert areas between the southern border of Jordan and the Jubbah basin. JQ-101 contributes to the growing number of new discoveries in Arabia, highlighting the significance of the archaeological and environmental records of the region. A key point to emerge here is the need to broaden Neolithic research beyond the borders of the Fertile Crescent.

## Materials and Methods

### Ethics

All necessary permits for the Jubbah fieldwork and analyses were obtained from the Saudi Commission for Tourism and Antiquities, Kingdom of Saudi Arabia.

## References

[1] Childe VG (1935) New light on the most ancient East: the oriental prelude to European prehistory. London: Kegan Paul/Trench/Trubner.

[2] Braidwood RJ, Howe B (1960) Prehistoric investigations in Iraqi Kurdistan. Studies in Ancient Oriental Civilization 31. Chicago: University of Chicago Press.

[3] Bar-Yosef O (1980) Prehistory of the Levant. Annu Rev Anthropol 9: 101–133.

[4] Bar-Yosef O (1981a) The “Pre-Pottery Neolithic” period in the southern Levant. In: Cauvin J, Sanlaville P, editors. Préhistoire du Levant. Paris: CNRS éditions. 555–569.

[5] Moore AMT (1982) Agricultural origins in the Near East, a model for the 1980’s. World Archaeol 14: 224–236.

[6] Hole F (1984) A reassessment of the Neolithic Revolution. Paléorient 10(2): 49–60.

[7] Aurenche O, Kozlowski SK (1999) La naissance du Néolithique au Proche-Orient. Paris: Editions Errance.

[8] Cauvin J (2000) The birth of the gods and the origins of agriculture. Cambridge: Cambridge University Press.

[9] Perrot J (2000) Réflexions sur l’état des recherches concernant la Préhistoire récente du Proche et du Moyen-Orient. Paléorient 26(1): 5–27.

[10] Wright GA (1971) Origins of food production in southwestern Asia: a survey of ideas. Curr Anthropol 12(4–5): 447–477.

[11] Bar-Yosef O, Meadow RH (1995) The origins of agriculture in the Near East. In: Price TD, Gebauer A-B, editors. Last hunters, first farmers: new perspectives on the prehistoric transition to agriculture. Santa Fe: School of American Research Press. 39–94.

[12] Damania A, Valkoun J, Willcox G, Qualset C (1999) The origins of agriculture and crop domestication. Aleppo: ICARDA.

[13] Zeder MA (2011a) The origins of agriculture in the Near East. Curr Anthropol 52(S4): S221–S235.

[14] Fuller D, Willcox G, Allaby R (2012) Early agricultural pathways: moving outside the “core area” hypothesis in Southwest Asia. J Exp Bot 63(2): 617–633. 22058404 10.1093/jxb/err307

[15] Willcox G, Nesbitt M, Bittmann F (2012) From collecting to cultivation: transitions to a production economy in the Near East (Editorial). Special Issue The Origins of agriculture in the Near East. Veg Hist Archaeobot 21(2): 81–83.

[16] Crabtree PJ (1993) Early animal domestication in the Middle East and Europe. Archaeological Method and Theory 5: 201–245.

[17] Horwitz LK, Tcherov E, Ducas P, Becker C, Von Den Driesch A, et al (1999) Animal domestication in the Southern Levant. Paléorient 25(2): 63–80.

[18] Zeder MA, Emshwiller E, Smith BD, Bradley DG (2006) Documenting domestication: the intersection of genetics and archaeology. Trends Genet 22: 139–155. 16458995 10.1016/j.tig.2006.01.007

[19] Zeder MA (2011b) Pathways to animal domestication. In: Damania A, Gepts P, editors. Harlan II: biodiversity in agriculture: domestication, evolution, and sustainability. Davis: University of California.

[20] Vigne J-D, Briois F, Zazzo A, Willcox G, Cucchi T, et al.. (2012) First wave of cultivators spread to Cyprus at least 10,600 y ago. Proc Natl Acad Sci U S A doi: 10.1073/Proc Natl Acad Sci U S A.1201693109. 10.1073/pnas.1201693109 PMC3365171 22566638

[21] Bar-Yosef O, Belfer-Cohen A (1992) From foraging to farming in the Mediterranean Levant. In: Gebauer AB, Price TD, editors. Transitions to agriculture in prehistory. Madison: Prehistory Press. 21–48.

[22] Finlayson B, Mithen SJ, Najjar M, Smith S, Maričević D, et al (2011) Architecture, sedentism, and social complexity at Pre-Pottery Neolithic A WF16, Southern Jordan. Proc Natl Acad Sci U S A 108(20): 8183–8188. 21536900 10.1073/pnas.1017642108 PMC3100919

[23] Scarre C (2005) The world transformed: from foragers and farmers to states and empires. In: Scarre C, editor. The Human Past: world prehistory and the development of human societies. London: Thames and Hudson. 183–193.

[24] Bocquet-Appel J-P, Bar-Yosef O (2008) The Neolithic demographic transition and its consequences. New York: Springer.

[25] Bocquet-Appel J-P (2011) When the world’s population took off: The springboard of the Neolithic demographic transition. Science 333(6042): 560–561. 21798934 10.1126/science.1208880

[26] Fernández E, Ortiz JE, Torres T, Pérez-Pérez A, Gamba C, et al (2008) Mitochondrial DNA genetic relationships at the ancient Neolithic site of Tell Halula. Forensic Sci Int Genet 1(1): 271–273.

[27] Diamond J, Bellwood P (2003) Farmers and their languages: the first expansions. Science 300(5619): 597–603. 12714734 10.1126/science.1078208

[28] Stordeur D (2003) Des crânes surmodelés à Tell Aswad de Damascène (PPNB - Syrie). Paléorient 29(2): 109–116.

[29] Banning EB (2011) So fair a house: Göbekli Tepe and the identification of temples in the Pre-Pottery Neolithic of the Near East. Curr Anthropol 52(5): 619–660.

[30] Hours F, Aurenche O, Cauvin J, Cauvin M-C, Copeland L, et al.. (1994) Atlas des sites du Proche-Orient (14000–5700 BP). Travaux de la Maison de l’Orient Méditerranéen No 24, Paris: de Boccard.

[31] Kenyon KM (1957) Digging up Jericho. London: Benn.

[32] Zohary D, Hopf M (1988) Domestication of plants in the old world: The origin and spread of cultivated plants in west Asia, Europe, and the Nile Valley. Oxford: Clarendon Press.

[33] Willcox G, Stordeur D (2012) Large-scale cereal processing before domestication during the tenth millennium cal BC in northern Syria. Antiquity 86 (331): 99–114.

[34] Helmer D (1989) Le développement de la domestication au Proche-Orient de 9500 à 7500 BP: les nouvelles données d’El Kowm et de Ras Shamra. Paléorient 15(1): 111–121.

[35] Zeder MA (2008) Domestication and early agriculture in the Mediterranean Basin: origins, diffusion, and impact. Proc Natl Acad Sci U S A 105: 11597–11604. 18697943 10.1073/pnas.0801317105 PMC2575338

[36] Kuijt I (1996) Negotiating equality through ritual: a consideration of Late Natufian and Prepottery Neolithic A period mortuary practices. Journal of Anthropological Archaeology 15(4): 313–336.

[37] Bocquentin F (2006) Pour une approche anthropologique de la transition Épipaléolithique-Néolithique au Proche-Orient. Bulletin du Centre de recherche français à Jérusalem 17: 41–51.

[38] Kuijt I (2000) People and space in early agricultural villages: exploring daily lives, community size and architecture in the Late Pre-Pottery Neolithic. Journal of Anthropological Archaeology 19: 75–102.

[39] Rollefson GO (2004) The character of LPPNB social organization. In: Bienert HD, Gebel HGK, Neef R, editors. Central settlements in Neolithic Jordan. Studies in Early Near Eastern production, subsistence, and environment 5. Berlin: ex oriente. 145–156.

[40] Abbo S, Lev-Yadun S, Gopher A (2010) Agricultural origins: centres and non-centres: a Near East reappraisal. CRC Crit Rev Plant Sci 29: 317–328.

[41] Cauvin J (1968) Fouilles de Byblos IV : les outillages néolithiques de Byblos et du littoral libanais. Paris: Maisonneuve.

[42] Gopher A (1994) Arrowheads of the Neolithic Levant: a seriation analysis, Dissertation Series 10, American Schools of Oriental Research. Winoma Lake: Eisenbraums.

[43] Quintero LA, Wilke PJ (1995) Evolution and economic significance of naviform Core-and-Blade technology in the southern Levant. Paléorient 21(1): 17–33.

[44] Nishiaki Y (2000) Lithic technology of Neolithic Syria. BAR International Series S840. Oxford: Archaeopress.

[45] Abbès F (2003) Les outillages néolithiques en Syrie du Nord, Méthode de débitage et gestion laminaire durant le PPNB. BAR International Series S1150. Oxford: Archaeopress.

[46] Arimura M (2003) The lithic production system in the Northwestern Levant from the LPPNB to the Early Pottery Neolithic: a view from Tell el-Kerkh. In: Iwasaki T. and Tsuneki A (eds.) Archaeology of the Rouj Basin: A Regional Study of the Transition from Village to City in Northwest Syria. Tsukuba: University of Tsukuba, Department of Archaeology, Institute of History and Anthropology. 155–165.

[47] Barzilai O (2010) Social complexity in the Southern Levantine PPNB as reflected through lithic studies, the bidirectional blade industries. BAR International Series S2180. Oxford: Archaeopress.

[48] Borrel F (2011) Bi-directional Neolithic blade technology in the northern Levant during the 7^th^–8^th^ millennia Cal B.C.: New insights from Mamarrul Nasr 2, Syria. Journal of Field Archaeology 36(2): 132–150.

[49] Rhotert H (1938) Transjordanien, Vorgeschichtliche, Forschungen. Stuttgart: Vorlag Strechker und Schröder.

[50] Adams R, Parr P, Ibrahim M, Al-Mughannum AS (1977) Saudi Arabian archaeological reconnaissance - 1976. Preliminary report on the first phase of the comprehensive survey archaeological survey programme. Atlal 1: 21–40.

[51] Parr PJ, Zarins J, Ibrahim M, Waechter J, Garrard A, et al (1978) Preliminary report on the second phase of the Northern Province survey 1397/1977. Atlal 2: 29–50.

[52] Inghraham M, Johnson T, Rihani B, Shatla I (1981) Preliminary report on a reconnaissance survey of the northwestern province (with a note on a brief survey on the northern province). Atlal 5: 59–84.

[53] Garrard AN, Harvey P, Switsur V (1981) Environment and settlement during the Upper Pleistocene and Holocene at Jubbah in the Great Nefud, northern Arabia. Atlal 5: 137–148.

[54] Gilmore M, Al-Ibrahim M, Murad AS (1982) Preliminary report on the Northwestern and Northern region survey 1981 (1401). Atlal 6: 9–23.

[55] Lehner B, Verdin K, Jarvis A (2008) New global hydrography derived from spaceborne elevation data. Eos 89(10): 93–94.

[56] Petraglia MD, Alsharekh A, Breeze P, Clarkson C, Crassard R, et al (2012) Hominin dispersal into the Nefud Desert and Middle Palaeolithic settlement along the Jubbah palaeolake, northern Arabia. PLoS One 7(11): e49840. 23185454 10.1371/journal.pone.0049840 PMC3501467

[57] Crassard R, Petraglia M, Drake N, Breeze P, Gratuze B, et al.. (2013). Middle Palaeolithic and Neolithic occupations around Mundafan palaeolake, Saudi Arabia: implications for climate change and human dispersals. PLoS One. In press. 10.1371/journal.pone.0069665 PMC3722113 23894519

[58] Petraglia MD, Alsharekh AM, Crassard R, Drake NA, Groucutt H, et al (2011) Middle Paleolithic occupation on a Marine Isotope Stage 5 lakeshore in the Nefud Desert, Saudi Arabia. Quat Sci Rev 30: 1555–1559.

[59] Khan M (2007) Rock-art of Saudi Arabia across twelve thousand years. Riyadh: Deputy Ministry of Antiquities and Museums.

[60] Jennings R, Shipton C, Al-Omari A, Alsharekh AM, Crassard R, et al.. (2013) A spatial rock-art survey of four jebels at Jubbah Oasis, Saudi Arabia. Antiquity. In press.

[61] Crassard R (2008) La Préhistoire du Yémen. Diffusions et diversités locales, à travers l’étude d’industries lithiques du Hadramawt. BAR International Series S1842. Oxford: Archaeopress.

[62] Goldberg P, Whitbread I (1993) Micromorphological study of a Bedouin tent floor. In: Goldberg P, Nash DT, Petraglia MD, editors. Formation processes in archaeological context. Madison, Wisconsin: Prehistory Press. 165–187.

[63] Dearing J (1999) Magnetic susceptibility. In: Walden J, Oldfield F, Smith J, editors. Environmental magnetism: a practical guide. Technical Guide 6. London: Quaternary Research Association. 35–63.

[64] Dean WE Jr (1974) Determination of carbonate and organic matter in calcareous sediments and sedimentary rocks by loss on ignition: Comparison with other methods. J Sediment Petrol 44: 242–248.

[65] Heiri O, Lotter AF, Lemcke G (2001) Loss on ignition as a method for estimating organic and carbonate content in sediments: reproducibility and comparability of results. J Paleolimnol 25: 101–110.

[66] Besler H, Ritter M (2009) Environmental histories of some Arabian Sands (Oman, United Arab Emirates) deduced from extended sedimentary analysis. Zeitschrift für Geomorphologie 53: 487–504.

[67] Huntley DJ, Godfrey-Smith DI, Thewalt MLW (1985) Optical dating of sediments. Nature 313: 105–107.

[68] Jacobs Z, Roberts RG (2007) Advances in optically stimulated luminescence dating of individual grains of quartz from archeological deposits. Evol Anthropol 16: 210–223.

[69] Jacobs Z, Hayes EH, Roberts RG, Galbraith RF, Henshilwood CS (2013) An improved OSL chronology for the Still Bay layers at Blombos Cave, South Africa: further tests of single-grain dating procedures and a re-evaluation of the timing of the Still Bay industry across southern Africa. J Archaeol Sci 40: 579–594.

[70] Galbraith RF, Roberts RG, Laslett GM, Yoshida H, Olley JM (1999) Optical dating of single and multiple grains of quartz from Jinmium rock shelter, northern Australia: Part I, experimental design and statistical models. Archaeometry 41: 339–364.

[71] Galbraith RF, Roberts RG (2012) Statistical aspects of equivalent dose and error calculation and display in OSL dating: an overview and some recommendations. Quat Geochronol 11: 1–27.

[72] Cauvin MC (1974) Flèches à encoches de Syrie, essai de classification et d’interprétation culturelle. Paléorient 2(2): 311–322.

[73] Kozlowski SK, Aurenche O (2005) Territories, boundaries and cultures in the Neolithic Near East. BAR International Series 1362. Oxford: Archaeopress.

[74] Neuville R, Bentor Y, Haas G, Perrot J, Vaufrey R (1951) Le Paléolithique et le Mésolithique du désert de Judée. Archives de l’Institut de Paléonthologie Humaine, Mémoire 24. Paris: Masson.

[75] Gonzalez-Echegaray JG (1964) Excavaciones en la Terraza de “El-Khiam” (Jordania), Vol.1. Madrid: Biblioteca Praehistórica Hispana.

[76] Gonzalez-Echegaray JG (1966) Excavaciones en la Terraza de “El-Khiam” (Jordania), Vol.2. Madrid: Biblioteca Praehistórica Hispana.

[77] Bar-Yosef O (1981b) Neolithic sites in Sinai. In: Wiesbaden: Tübinger Atlas des Vorderen Orients (TAVO) A Frey W, Uerpmann H-P, editors. Beiträge zur Umweltgeschichte des Vorderen Orients. 8: 217–235.

[78] Perrot J (1952) Têtes de flèches du Natoufien et du Tahounien (Palestine). Bulletin de la Société Préhistorique Française 49(8): 439–449.

[79] Garfinkel Y, Nadel D (1989) The Sultanian flint assemblage from Gesher and its implications for recognizing Early Neolithic entities in the Levant. Paléorient 15(2): 139–151.

[80] Cauvin MC, Abbès F (2008) Analyse du mobilier retouché. In: Ibañez JJ, editor. Le site néolithique de Tell Mureybet (Syrie du Nord), Vol I. BAR International Series S1843.

[81] Yartah T (2001) Emmanchement de pointes de flèches khiamiennes de Syrie du Nord. In: Bourguignon L, Ortega I, Frère-Sautot M-C, editors. Préhistoire et approche expérimentale. Montagnac: éditions Monique Mergoil. 281–292.

[82] Aurenche O, Kozlowski SK (2011) The spatial distribution of arrowheads and microliths in the Near East (10,200–8,000 cal. BC). In: Healey E, Campbell S, Maeda O, editors. The state of the stone: terminologies, continuities and contexts in Near Eastern lithics. Studies in Early Near Eastern Production, Subsistence, and Environment 13. Berlin: ex oriente. 449–456.

[83] Gopher A (1989) Diffusion process in the Pre-Pottery Neolithic Levant: the case of the Helwan point. In: Hershkovitz I, editor. People and culture in change. BAR International Series S508. Oxford: Archaeopress. 91–106.

[84] Mortensen P (1970) A preliminary study of the chipped stone industry from Beidha, an Early Neolithic village in Southern Jordan. Acta Archaeologica 41(1): 1–54.

[85] Rosen SA (1997) Lithics after the Stone Age. A handbook of stone tools from the Levant. Walnut Creek: Altamira press.

[86] Drechsler P (2009) The Dispersal of the Neolithic over the Arabian Peninsula. BAR International Series S1969. Oxford: Archaeopress.

[87] Charpentier V (2004) Trihedral points: a new facet to the “Arabian Bifacial Tradition”. Proceedings of the Seminar for Arabian Studies 34: 53–66.

[88] Crassard R (2009) Modalities and characteristics of human occupations in Yemen during the Early/Mid-Holocene. C.R. Geoscience 341: 713–725.

[89] Waechter Jd’A, Seton-Williams VM (1938) The excavations at Wadi Dhobai 1937–1938 and the Dhobaian industry. The Journal of the Palestine Oriental Society 18: 172–186.

[90] Field H (1960) North Arabian desert archaeological survey, 1925–50. Papers of the Peabody Museum of Archaeology and Ethnology, Harvard University, Vol. 45, No. 2.

[91] Betts A (1982) Prehistoric sites at Qa’a Mejalla, Eastern Jordan. Levant (14): 1–34.

[92] Abe Y, Akazawa T (1977) Burin factory site, Palmyra, Syria. Bulletin of the National Science Museum, Series D (Anthropology), Vol. 3: 1–22.

[93] Cauvin M-C, Cauvin J (1993) La Séquence néolithique PPNB au Levant Nord. Paléorient 19(1): 23–28.

[94] MacDonald E (1932) Prehistoric Fara. In: MacDonald E, Starkey JL, Harding L, editors. Beth-Pelet II. London: British School of Archaeology in Egypt: 1–21.

[95] Bar-Yosef O, Gopher A, Nadel D (1987) The “Hagdud Truncation” - A new type from the Sultanian Industry at Netiv Hagdud, Jordan Valley. Mitekufat Haeven, Journal of the Israel Prehistoric Society 20: 151–157.

[96] Noy T (1994) Gilgal Truncation. A new type of tool from Early PPNB. In: HG Gebel, SK Kozlowski, editors. Neolithic Chipped Stone Industries of the Fertile Crescent. Berlin: ex oriente. 423–426.

[97] Goudie AS, Colls A, Stokes S, Parker AG, White K, et al (2000) Late Pleistocene and Holocene dune construction at the north eastern edge of the Rub Al Khali, United Arab Emirates. Sedimentology 47: 1011–1021.

[98] Preusser F (2009) Chronology of the impact of Quaternary climate change on continental environments in the Arabian Peninsula. C. R. Geoscience 341: 621–632.

[99] Engel M, Brückner H, Pint A, Wellbrock K, Ginau A, et al (2012) The early Holocene humid period in NW Saudi Arabia - Sediments, microfossils and palaeo-hydrological modeling. Quat Int 266: 131–141.

[100] Whitney JW, Faulkender DJ, Rubin M (1983) The environmental history and present condition of Saudi Arabia’s northern sand seas. USGS Open File Report 83–749.

[101] Schulz E, Whitney JW (1986) Upper Pleistocene and Holocene lakes in the An Nefud, Saudi Arabia. Hydrobiologia 143: 175–190.

[102] McClure HA (1976) Radiocarbon chronology of late Quaternary lakes in the Arabian Desert. Nature 263: 755–756.

[103] Rosenberg TM, Preusser F, Fleitmann D, Schwalb A, Penkman K, et al (2011) Humid periods in southern Arabia: Windows of opportunity for modern human dispersal. Geology 39: 1115–1118.

[104] Fleitmann D, Burns SJ, Pekala M, Mangini A, Al-Subbary A, et al (2011) Holocene and Pleistocene pluvial periods in Yemen, southern Arabia. Quat Sci Rev 30: 783–787.

[105] Radies D, Hasiotis ST, Preusser F, Neubert E, Matter A (2005) Paleoclimatic significance of Early Holocene faunal assemblages in wet interdune deposits of the Wahiba Sand Sea, Sultanate of Oman. J Arid Environ 62: 109–125.

[106] Parker AG, Eckersley L, Smith MM, Goudie AS, Stokes S, et al (2004) Holocene vegetation dynamics in the northeastern Rub’ al- Khali desert, Arabian Peninsula: a phytolith, pollen and carbon isotope study. Journal of Quaternary Science 19: 665–676.

[107] Arz HW, Lamy F, Patzold J, Muller PJ, Prins M (2003) Mediterranean Moisture Source for an Early-Holocene Humid Period in the Northern Red Sea. Science 300: 118–121. 12677064 10.1126/science.1080325

[108] Charpentier V (2008) Hunter-gatherers of the “empty quarter of the early Holocene” to the last Neolithic societies: chronology of the late prehistory of south-eastern Arabia (8000–3100 BC). Proceedings of the Seminar for Arabian Studies 38: 59–82.

[109] Kapel H (1967) Atlas of the Stone-Age Cultures of Qatar. Aarhus: Aarhus University Press.

[110] Crassard R, Drechsler P (2013) Towards new paradigms: multiple pathways for the Arabian Neolithic. Arabian Archaeology and Epigraphy 24 (1): 3–8.

[111] Groucutt HS, Petraglia MD (2012) The prehistory of the Arabian peninsula: deserts, dispersals, and demography. Evol Anthropol 21: 113–125. 22718479 10.1002/evan.21308

[112] Rose JI, Černý V, Bayoumi R (2013) Tabula rasa or refugia? Using genetic data to assess the peopling of Arabia. Arabian Archaeology and Epigraphy 24 (1) 95–101.

[113] Inizan M-L (1980) Sur les industries à lames de Qatar. Paléorient 6: 233–236.

[114] Inizan M-L (1988) Préhistoire à Qatar, vol. 2, Paris: Editions Recherche sur les Civilisations.

[115] Pélegrin J, Inizan M-L (2013) Soft hammerstone percussion use in bidirectional blade-tool production at Acila 36 and in bifacial knapping at Shagra (Qatar). Arabian Archaeology and Epigraphy 24 (1): 79–86.

[116] Charpentier V, Crassard R (2013) Back to Fasad… and the PPNB controversy. Questioning a Levantine origin for Arabian Early Holocene projectile points technology. Arabian Archaeology and Epigraphy 24 (1): 28–36.

[117] Rose JI, Petraglia MD (2009) Tracking the origin and evolution of human populations in Arabia. In: Petraglia MD, Rose JI, editors. The evolution of human populations in Arabia. Dordrecht: Springer. 1–12.

[118] Rose JI (2010) New light on human prehistory in the Arabo-Persian Gulf Oasis. Curr Anthropol 51: 849–883.

[119] Fedele FG (2009) Early Holocene in the highlands: Data on the peopling of the eastern Yemen plateau, with a note on the Pleistocene evidence. In: Petraglia MD, Rose JI, editors. The evolution of human populations in Arabia. Dordrecht: Springer. 215–236.

[120] Hilbert Y, Rose J, Roberts RG (2012) Late Palaeolithic core-reduction strategies in Dhofar, Oman. Proceedings of the Seminar for Arabian Studies 42: 101–118.

[121] Uerpmann H-P, Potts DT, Uerpmann M (2009) Holocene (re-)occupation of eastern Arabia. In: Petraglia MD, Rose JI, editors. The evolution of human populations in Arabia. Dordrecht: Springer. 205–214.

[122] Fernandes V, Alshamali F, Alves M, Costa MD, Pereira JB, et al (2012) The Arabian cradle: mitochondrial relicts of the first steps along the southern route out of Africa. Am J Hum Genet 90: 1–9. 10.1016/j.ajhg.2011.12.010 PMC3276663 22284828

[123] Al-Abri A, Podgorná E, Rose JI, Pereira L, Mulligan CJ, et al (2012) Pleistocene-Holocene boundary in southern Arabia from the perspective of human mtDNA variation. Am J Phys Anthropol 149(2): 291–8. 22927010 10.1002/ajpa.22131

[124] Jarvis A, Reuter HI, Nelson A, Guervara E (2008) Hole-filled seamless SRTM data V4, International Centre for Tropical Agriculture (CIAT), available from http://srtm.csi.cgiar.org.Accessed 2013 May 31.

[125] Prescott JR, Hutton JT (1994) Cosmic ray contributions to dose rates for luminescence and ESR dating: large depths and long-term time variations. Radiat Meas 23: 497–500.

[126] Powell R, Hergt J, Woodhead J (2002) Improving isochron calculations with robust statistics and the bootstrap. Chem Geol 185: 191–204.

[127] Reimer PJ, Baillie MGL, Bard E, Bayliss A, Beck JW, et al (2009) IntCal09 and Marine09 radiocarbon age calibration curves, 0–50,000 years cal BP. Radiocarbon 51: 1111–1150.

